# Supplementary material for: Structures of LPOR–Chlide complexes reveal the structural basis of membrane remodeling and photocatalysis
Source: Nat Commun. 2026 Aug 5;17:9427. doi: 10.1038/s41467-026-76316-7 (PMC13538550; doi:10.1038/s41467-026-76316-7)
Supplement: Supplementary file 1 [file 41467_2026_76316_MOESM1_ESM.pdf]

Supplementary information for

Structures of LPOR–Chlide complexes reveal the structural basis of membrane remodeling and photocatalysis

Michał Gabruk[1*], Ambroise Desfosses[2], Leandro Farias Estrozi[2], Sebastian Pintscher[3], Michał Rawski[4], Grzegorz Ważny[4], Agnieszka Garbacz[1], Mateusz Zbyradowski[1,5], Jerzy Kruk[1], Leszek Fiedor[1]

[1] Department of Plant Physiology and Biochemistry, Faculty of Biochemistry, Biophysics and Biotechnology, Jagiellonian University, Gronostajowa 7, 30-387 Kraków, Poland.

[2] Institut de Biologie Structurale, Université Grenoble Alpes, CEA, CNRS, IBS, Grenoble, France

[3] Faculty of Biochemistry, Biophysics and Biotechnology, Department of Plant Biotechnology, Jagiellonian University, Gronostajowa 7, 30-387 Kraków, Poland.

[4] SOLARIS National Synchrotron Radiation Centre, Jagiellonian University, Krakow, Poland

5 Present address: W. Szafer Institute of Botany, Polish Academy of Sciences, ul. Lubicz 46, 31-512 Kraków, Poland

*Corresponding author: Michal Gabruk (michal.gabruk@uj.edu.pl)

Supplementary Methods

Protein expression and purification

Competent Escherichia coli BL21(DE3) pRIL cells were transformed using the heat shock method and plated on LB agar supplemented with ampicillin (100 mg/L) and chloramphenicol (25 mg/L). Plates were incubated overnight at 37 °C.

A single colony was used to inoculate 50 mL of LB medium containing the same antibiotics, and the culture was grown overnight at 37 °C with shaking. The following day, 25 mL of the overnight culture was transferred into 1 L of LB medium supplemented with antibiotics and incubated at 37 °C with shaking for approximately 3 hours, until the OD_{600} reached ~0.6.

Protein expression was induced by the addition of IPTG to a final concentration of 0.5 mM, and the culture was incubated overnight at 22 °C with shaking. Cells were harvested by centrifugation, and the resulting pellet was frozen at −20 °C for further use.

For purification, the cell pellet was resuspended in phosphate buffer (50 mM Na_2HPO pH 7.1, 300 mM NaCl , 7 mM β-mercaptoethanol) supplemented with 1 mM PMSF. Cells were lysed by sonication (7 s on / 14 s off cycles for a total sonication time of 10 min). The lysate was clarified by centrifugation at 20,000 × g for 1 hour.

The supernatant was incubated with TALON metal affinity resin for 2 hours at 4 °C with gentle mixing. The resin was washed twice with the phosphate buffer, and bound proteins were eluted using phosphate buffer supplemented with 200 mM imidazole (using Amicon Pro tubes).

Fractions containing the target protein were pooled, and glycerol was added to a final concentration of 25% (v/v). Protein concentration was determined using the Bradford assay. The purified protein was aliquoted (1 mL), flash-frozen, and stored at −20 °C until further use.

TEM Sample preparation and negative staining

LPOR (30 µM; 70 µL) was mixed with 30 µL of 0.5 M arginine in the phosphate buffer (pH 7.1). NADPH and OPT lipids were added and the sample was immediately vortexed. "OPT lipids" denotes the lipid mixture optimized in our previous structural work[1], comprising 50 mol% MGDG, 35 mol% DGDG, and 15 mol% PG (Avanti Polar Lipids). All subsequent steps were performed under dim green light.

Chlorophyllide or protochlorophyllide in methanol stock solution was then added to a final concentration of 5 µM and the sample was vortexed briefly (~1 s). Samples were incubated for 7 minutes in darkness and subsequently centrifuged at 13,400 rpm for 7 minutes using a bench-top centrifuge. The supernatant was discarded, and the pellet was immediately resuspended in 10 µL of the phosphate buffer supplemented with 0.5 M arginine.

For grid preparation, 7 μL of the sample was applied onto a carbon-coated copper grid (FCF300-Cu, formvar/carbon, 300 mesh) and incubated for 5 minutes. The grid was then blotted and immediately stained with 2% (w/v) uranyl acetate (UA).

Staining was performed by sequential immersion of the grid (sample side facing down) into water and UA droplets: first for 1 s in water, then for 1 s in UA, followed by blotting, then immersion for 5 s in UA, and subsequently for 30 s in UA, each time in fresh droplets of stain. After the final blotting step, the grid was air-dried for approximately 1 minute.

Pchlide purification

Protochlorophyllide (Pchlide) was isolated from etiolated wheat seedlings as described previously[2,3], with additional purification steps to reduce lipid and carotenoid contamination. Briefly, dark-grown 8-day-old wheat seedlings were harvested and extracted with acetone under dim green light. The combined extracts were subjected to phase separation using chloroform and aqueous buffer (10 mM Tris-HCl buffer, pH 7.0, 5% NaCl), and the chloroform fractions containing pigments were collected, washed, and concentrated.

Pchlide was separated from carotenoids on Sephadex LH-20 column in methanol and finally purified using reverse-phase HPLC (Acclaim C30 column, Thermo Scientific) in acetonitrile/methanol/water (72/8/1, v/v), with detection at 435 nm (absorbance) and 432/640 nm (fluorescence).

All the procedures were performed under dim green light.

Chlide production

Chlorophyllide a (Chlide a) in stereochemically pure form (the non-prime diastereoisomer) was prepared from chlorophyll a (Chl a) by enzymatic removal of the phytyl chain using chlorophyllase from A. thaliana., expressed heterogenically in E. coli, as described previously[4]. Briefly, the AtCLH2 gene coding for Chlase was isolated from wild-type A. thaliana, amplified using RTPCR and cloned into a pGEX4T1 plasmid expression vector (Amersham Biosciences). AtCLH2 was expressed in E. coli BL21 strain and the reaction was carried in aqueous acetone, using the enzyme immobilized in the bacterial acetone powder. The product was purified by column chromatography on CM-Sepharose CL-6B, following a described previously protocol[5].

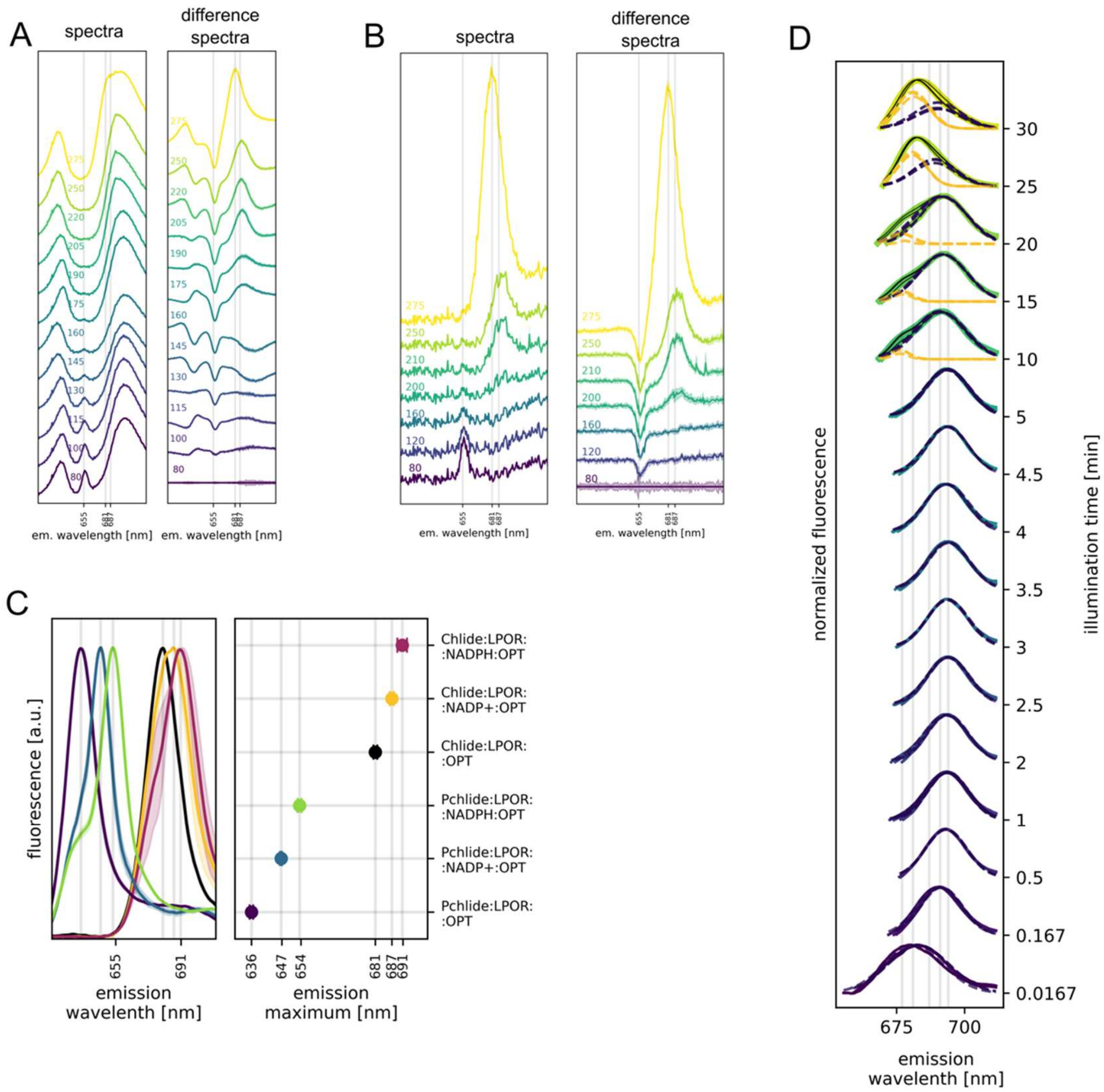

Supplementary Figure 1. Chlide:LPOR:NADPH complexes in vivo and in vitro. AB. Fluorescence spectra of wheat seedlings illuminated over a temperature range. Shaded areas represent standard deviation between replicates. On A, peak at ~720nm originates form a light contamination. These are replicates of the experiment shown in Fig. 1A (bottom panel). **C.** The fluorescence emission spectra of in vitro reconstituted complexes of LPOR (15μM) in complex with NADPH/NADP+ (200 μM) and Pchlide/Chlide (5 μM), in the presence of OPT lipids (400 μM), with emission maxima corresponding to complexes differing in their bound dinucleotide (NADPH or $NADP^+$) and pigment (Pchlide/Chlide). **D.** Difference fluorescence spectra of wheat seedlings (black) illuminated for varying time intervals and measured at 77 K with Gausian deconvolution. The experimental spectra are shown as solid lines in a violet-green–yellow color scale, where the color reflects illumination time. Dashed violet and yellow lines represent the two Gaussian components fitted to the data (just violet dashed line is shown then one Gaussian compotent was fitted), and the solid black line corresponds to their sum. Shaded area represents standard deviation between biological replicates.

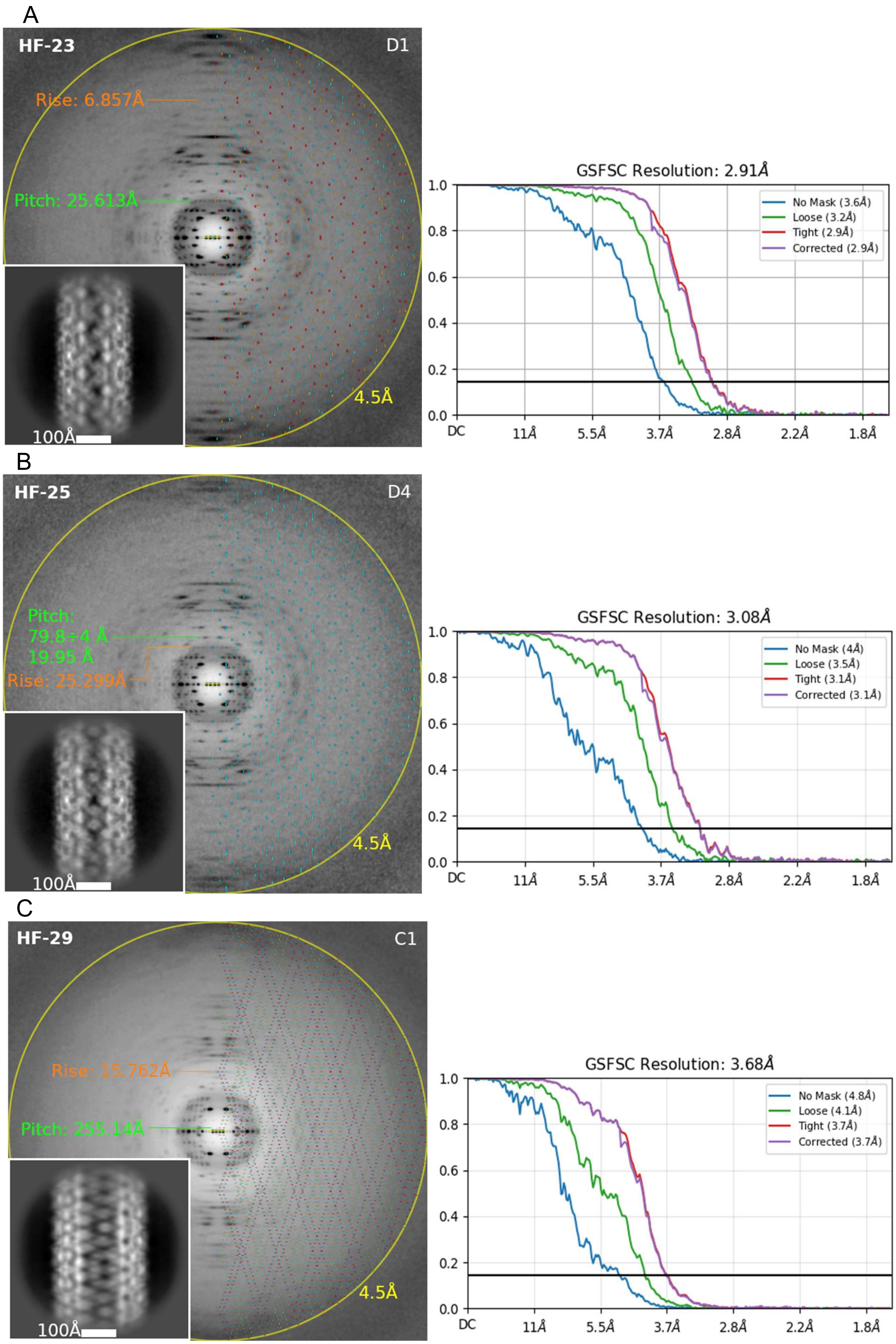
A
HF-23
D1
Rise: 6.857Å
Pitch: 25.613Å
4.5Å
100Å
GSFSC Resolution: 2.91Å
No Mask (3.6Å)
Loose (3.2Å)
Tight (2.9Å)
Corrected (2.9Å)
1.0
0.8
0.6
0.4
0.2
0.0
DC
11Å
5.5Å
3.7Å
2.8Å
2.2Å
1.8Å
B
HF-25
D4
Pitch:
79.8÷4 Å
19.95 Å
Rise: 25.299Å
4.5Å
100Å
GSFSC Resolution: 3.08Å
No Mask (4Å)
Loose (3.5Å)
Tight (3.1Å)
Corrected (3.1Å)
C
HF-29
C1
Rise: 15.762Å
Pitch: 255.14Å
4.5Å
100Å
GSFSC Resolution: 3.68Å
No Mask (4.8Å)
Loose (4.1Å)
Tight (3.7Å)
Corrected (3.7Å)

D

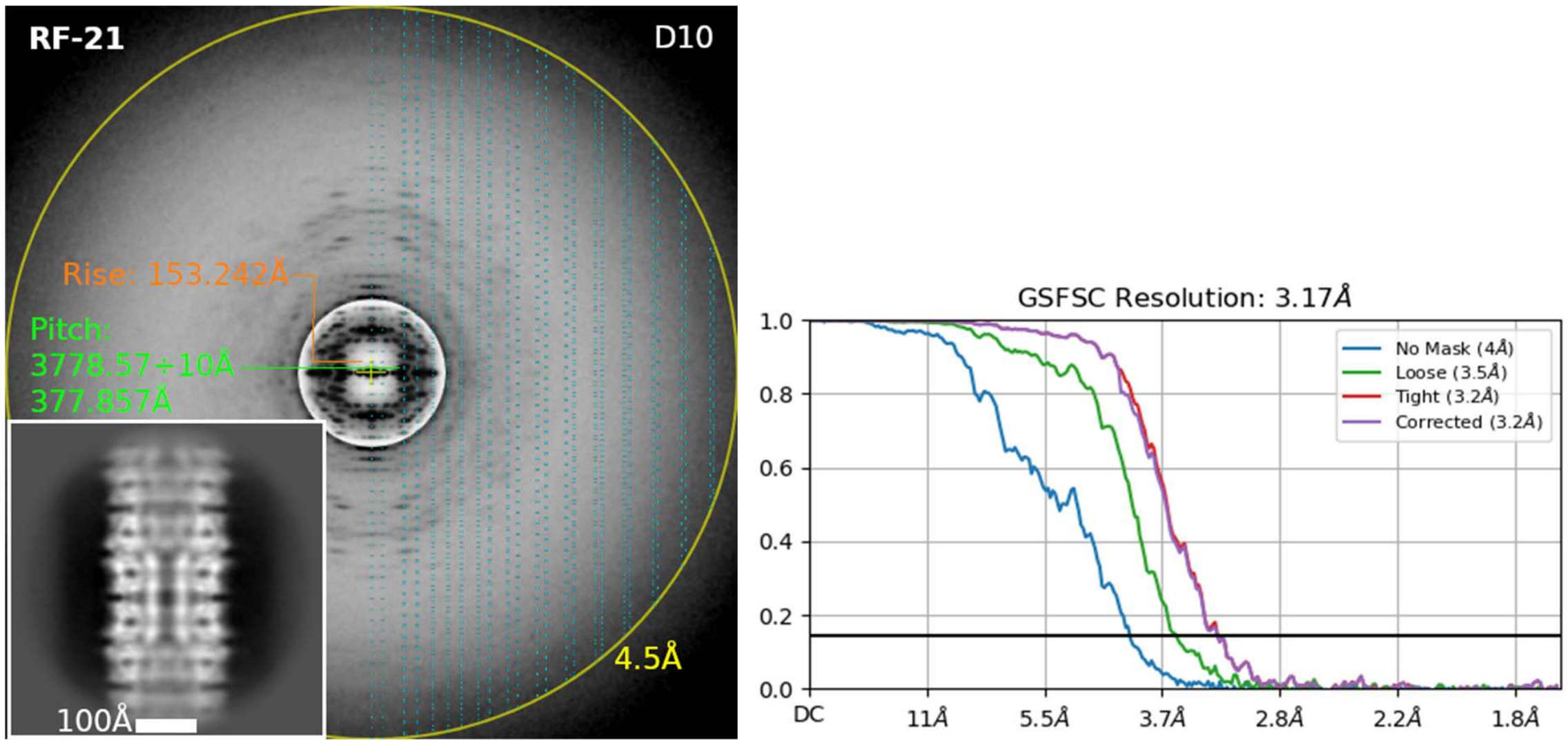
RF-21
D10
Rise: 153.242Å
Pitch:
3778.57÷10Å
377.857Å
4.5Å
100Å
GSFSC Resolution: 3.17Å
No Mask (4Å)
Loose (3.5Å)
Tight (3.2Å)
Corrected (3.2Å)
1.0
0.8
0.6
0.4
0.2
0.0
DC
11Å
5.5Å
3.7Å
2.8Å
2.2Å
1.8Å

E

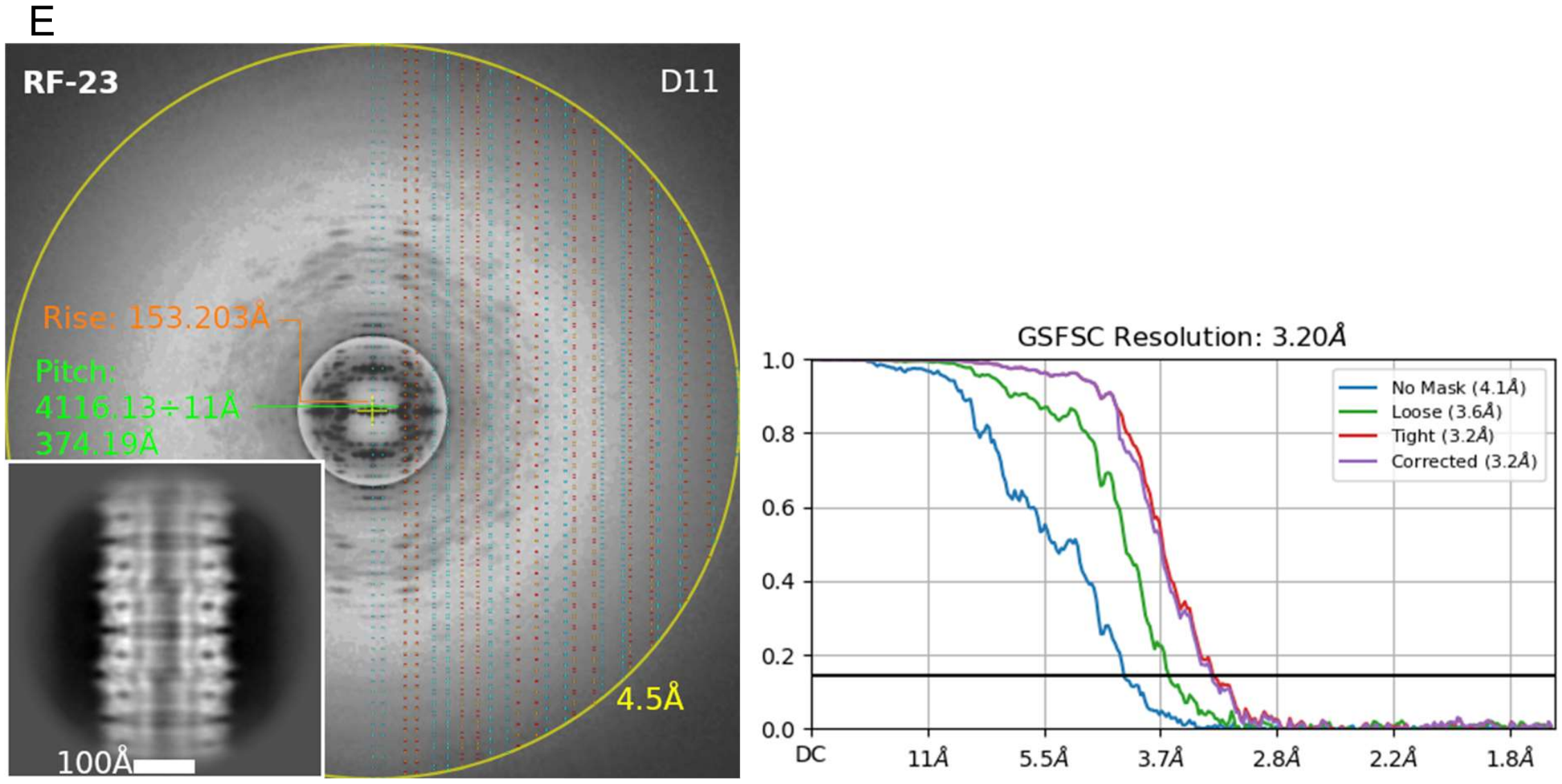
RF-23
D11
Rise: 153.203Å
Pitch:
4116.13÷11Å
374.19Å
4.5Å
100Å
GSFSC Resolution: 3.20Å
No Mask (4.1Å)
Loose (3.6Å)
Tight (3.2Å)
Corrected (3.2Å)
1.0
0.8
0.6
0.4
0.2
0.0
DC
11Å
5.5Å
3.7Å
2.8Å
2.2Å
1.8Å

F

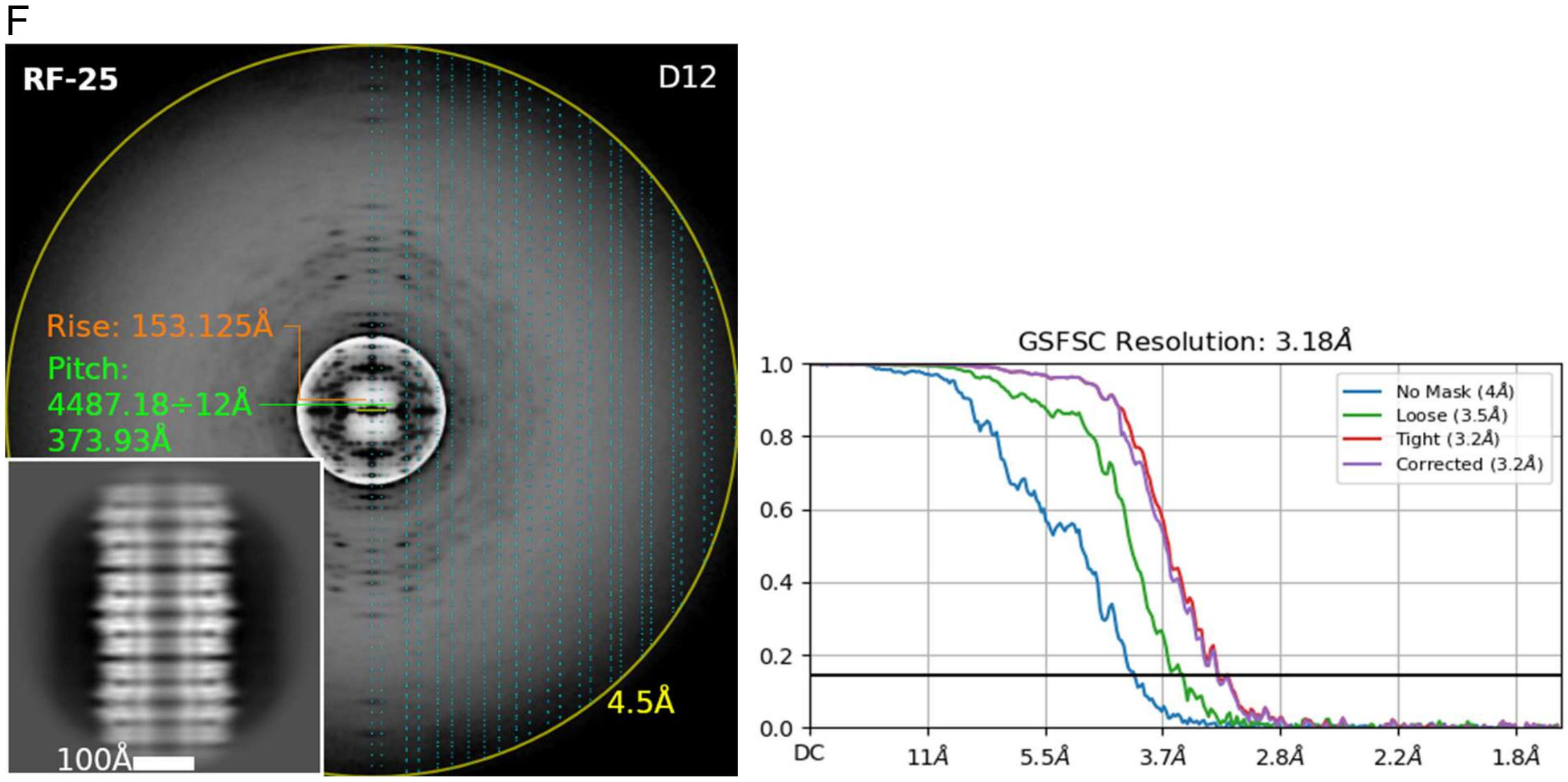
RF-25
D12
Rise: 153.125Å
Pitch:
4487.18÷12Å
373.93Å
4.5Å
100Å
GSFSC Resolution: 3.18Å
No Mask (4Å)
Loose (3.5Å)
Tight (3.2Å)
Corrected (3.2Å)
1.0
0.8
0.6
0.4
0.2
0.0
DC
11Å
5.5Å
3.7Å
2.8Å
2.2Å
1.8Å

G

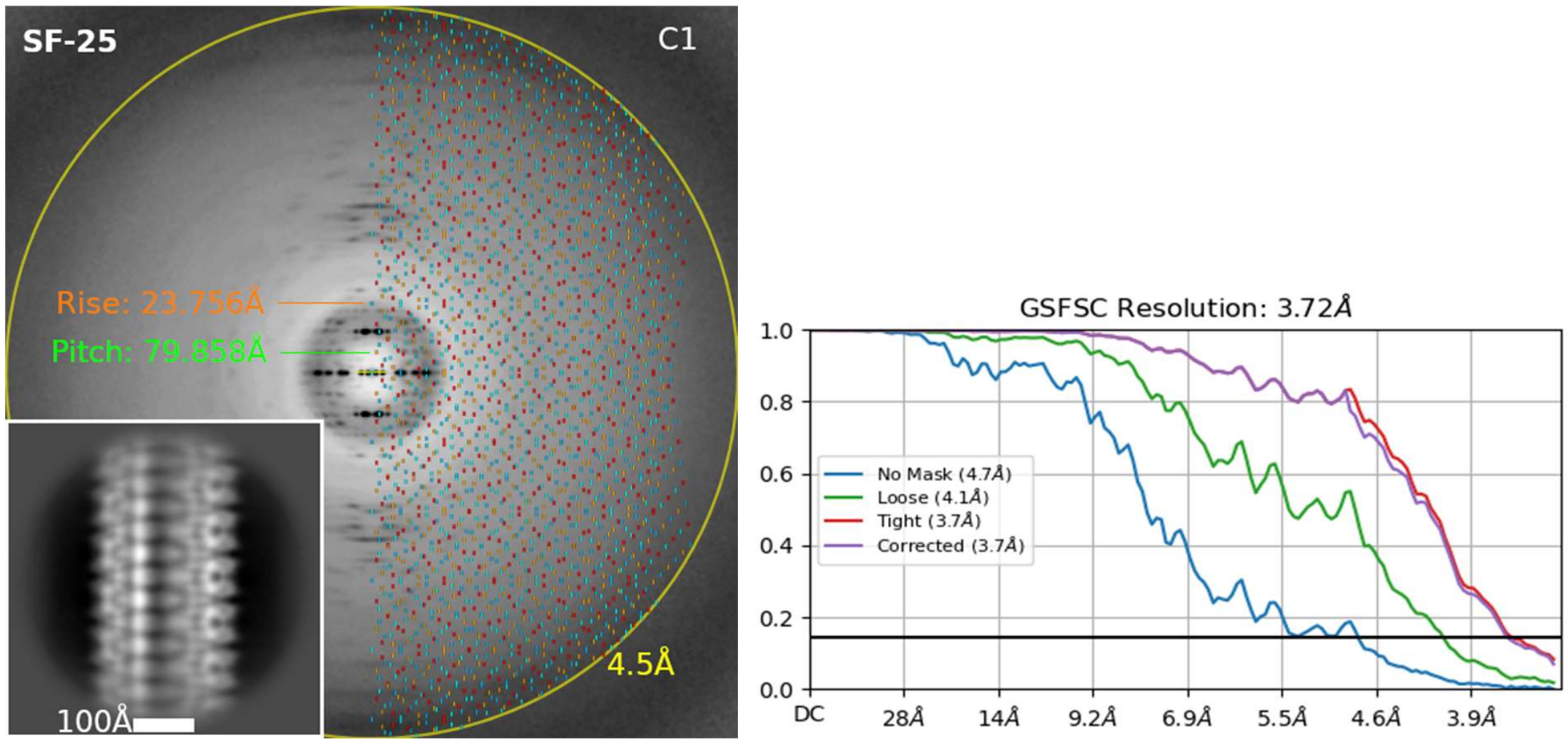
SF-25
C1
Rise: 23.756Å
Pitch: 79.858Å
4.5Å
100Å
GSFSC Resolution: 3.72Å
No Mask (4.7Å)
Loose (4.1Å)
Tight (3.7Å)
Corrected (3.7Å)
1.0
0.8
0.6
0.4
0.2
0.0
DC
28Å
14Å
9.2Å
6.9Å
5.5Å
4.6Å
3.9Å

H

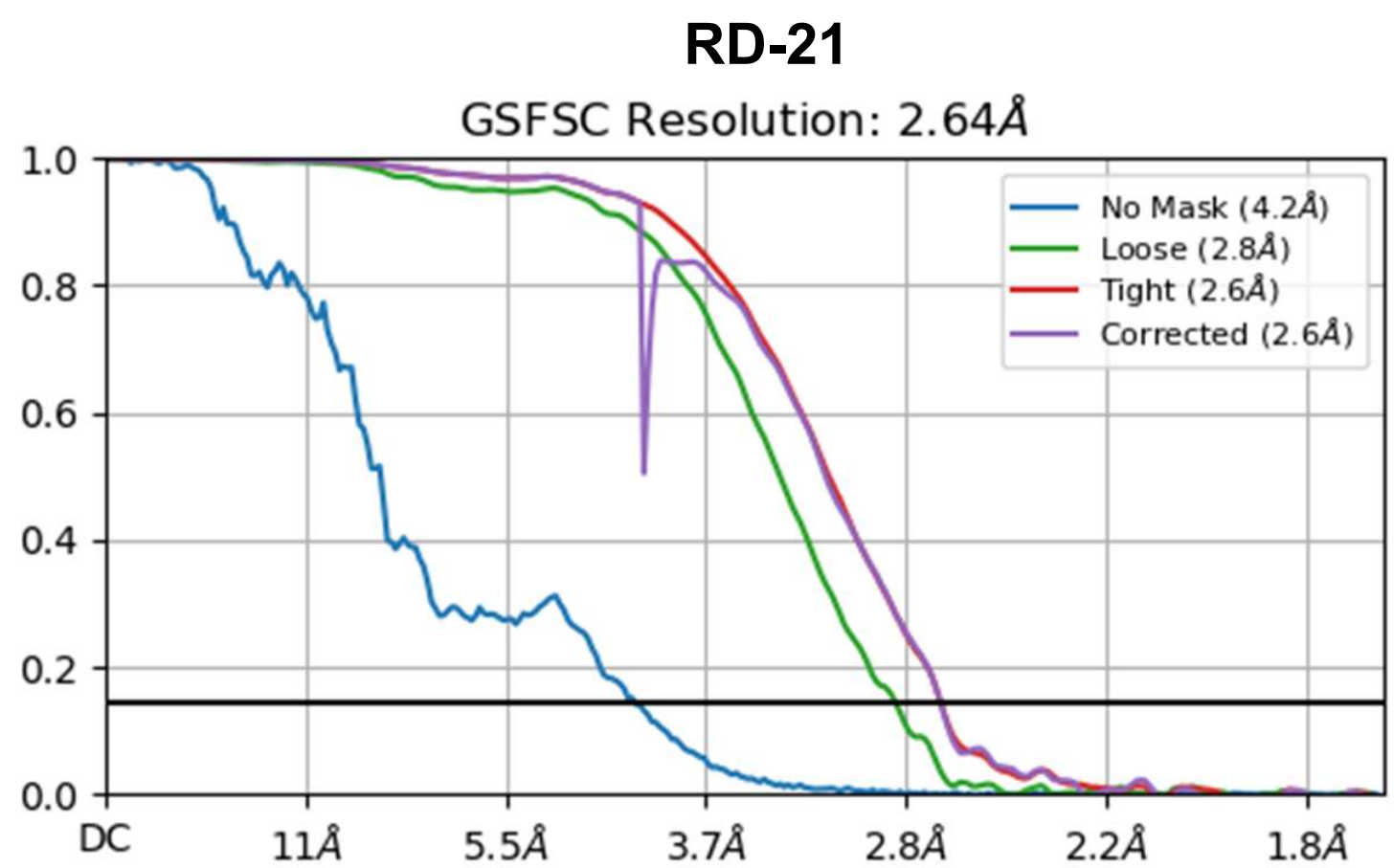
RD-21
GSFSC Resolution: 2.64Å
No Mask (4.2Å)
Loose (2.8Å)
Tight (2.6Å)
Corrected (2.6Å)
1.0
0.8
0.6
0.4
0.2
0.0
DC
11Å
5.5Å
3.7Å
2.8Å
2.2Å
1.8Å

I

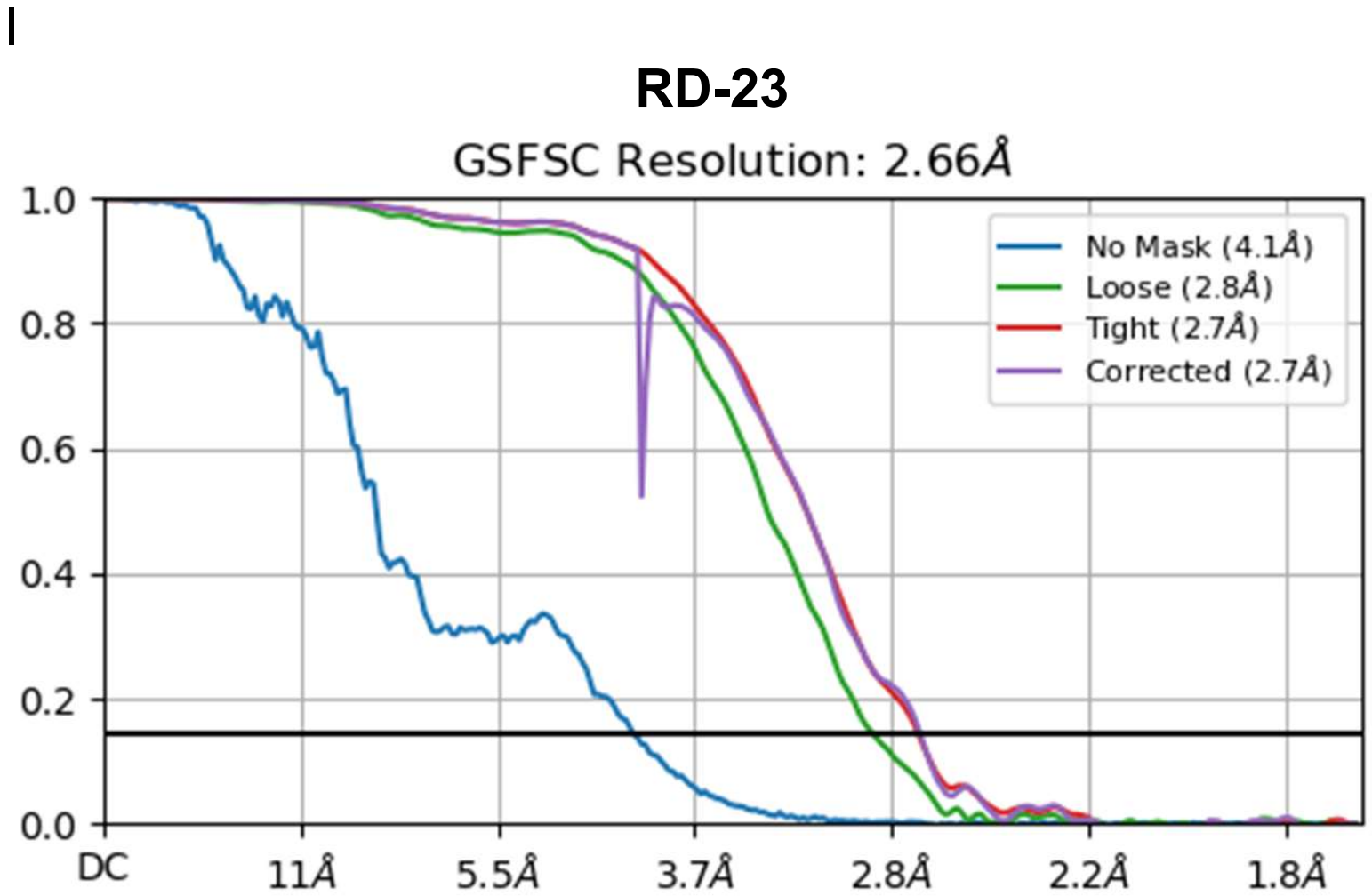
RD-23
GSFSC Resolution: 2.66Å
No Mask (4.1Å)
Loose (2.8Å)
Tight (2.7Å)
Corrected (2.7Å)
1.0
0.8
0.6
0.4
0.2
0.0
DC
11Å
5.5Å
3.7Å
2.8Å
2.2Å
1.8Å

J

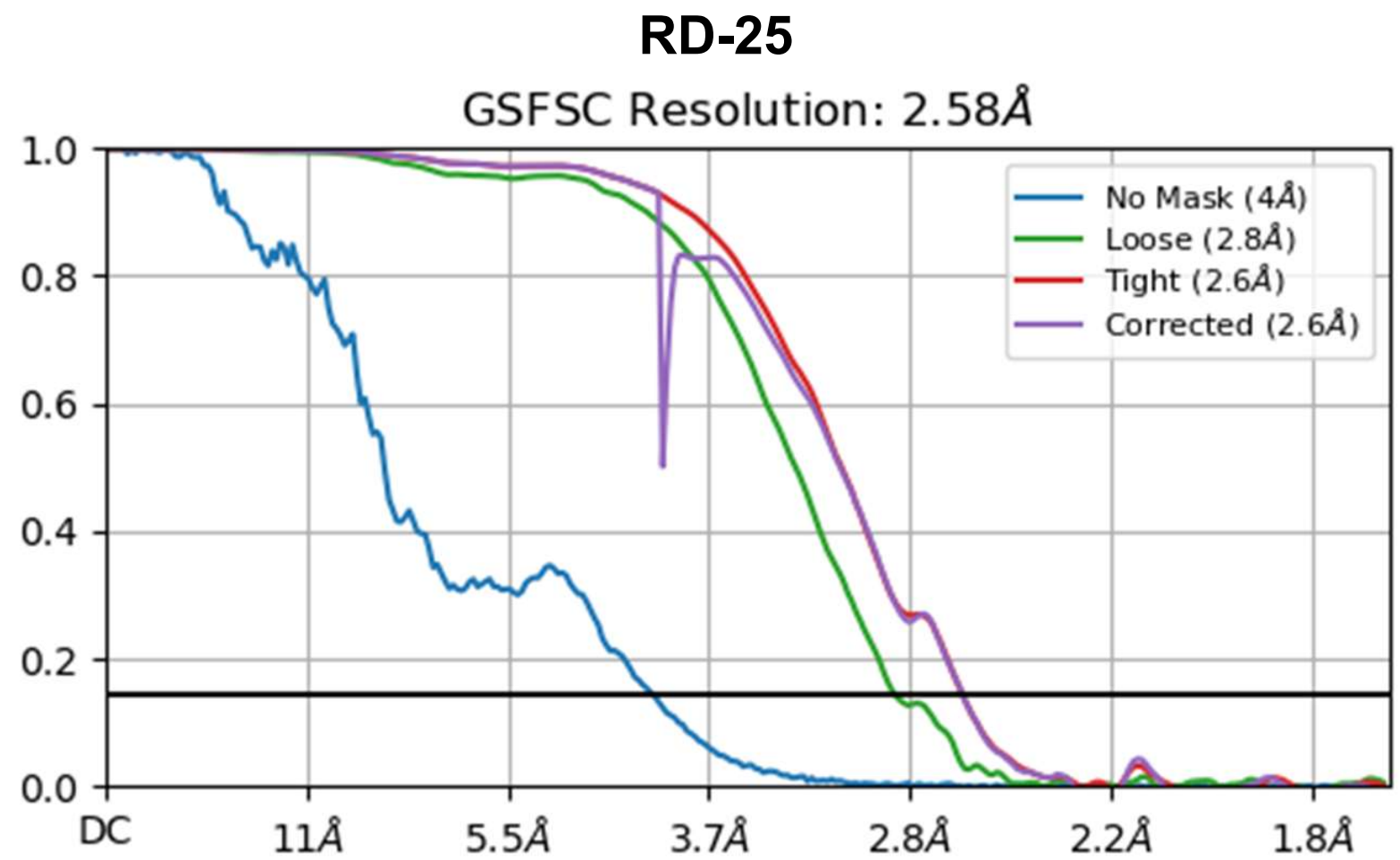
RD-25
GSFSC Resolution: 2.58Å
No Mask (4Å)
Loose (2.8Å)
Tight (2.6Å)
Corrected (2.6Å)
1.0
0.8
0.6
0.4
0.2
0.0
DC
11Å
5.5Å
3.7Å
2.8Å
2.2Å
1.8Å

K

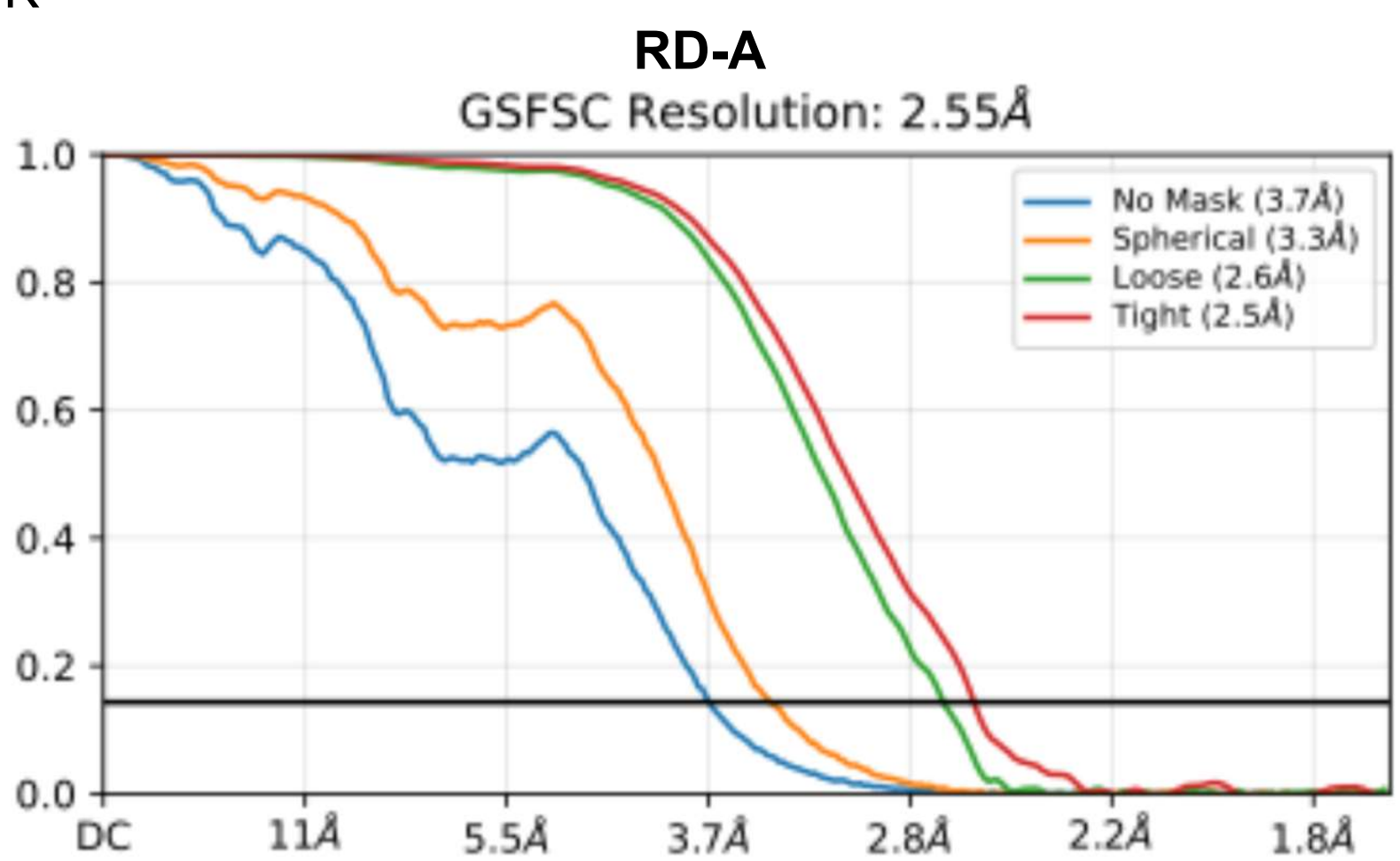
RD-A
GSFSC Resolution: 2.55Å
No Mask (3.7Å)
Spherical (3.3Å)
Loose (2.6Å)
Tight (2.5Å)
1.0
0.8
0.6
0.4
0.2
0.0
DC
11Å
5.5Å
3.7Å
2.8Å
2.2Å
1.8Å

L

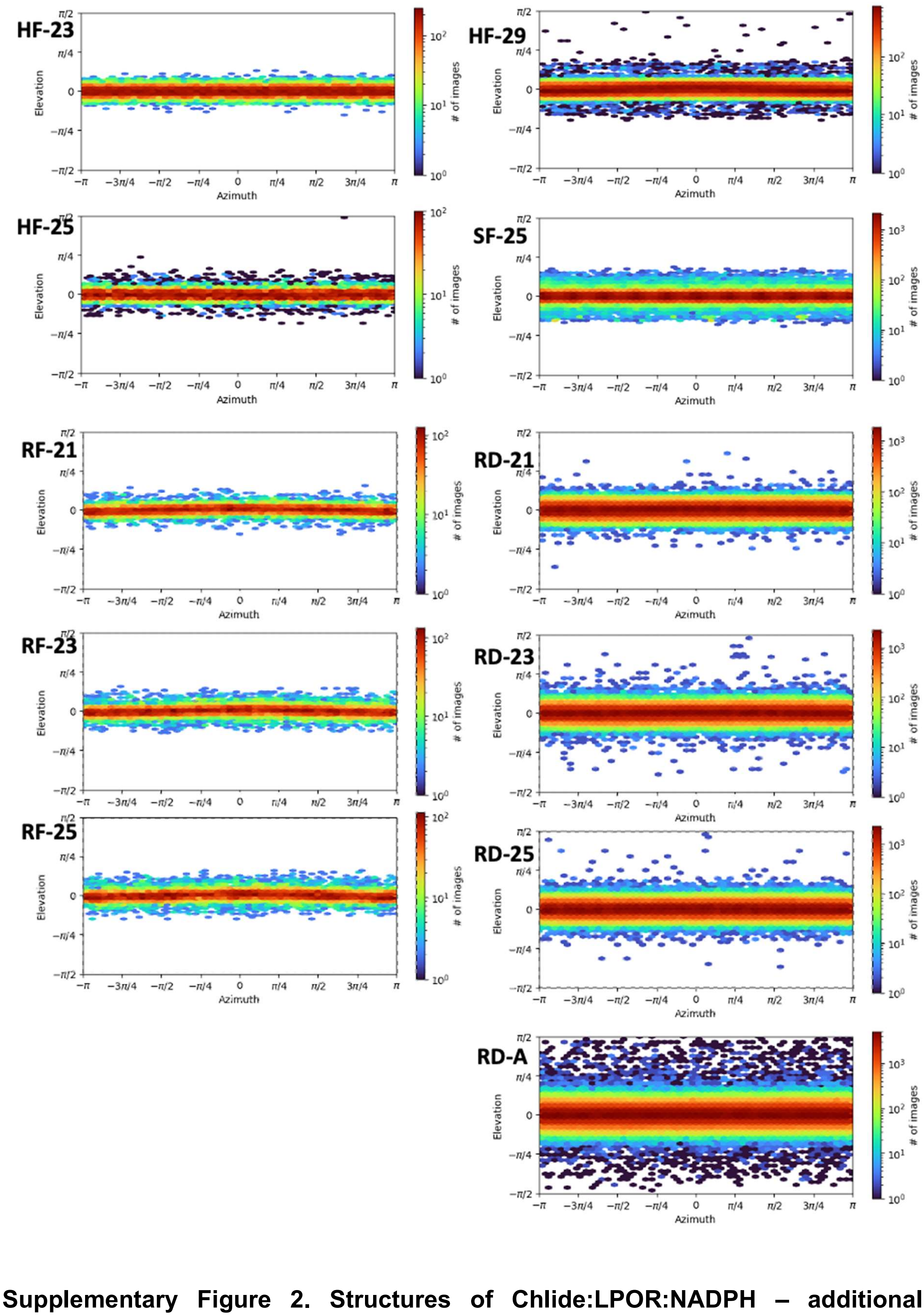

Supplementary Figure 2. Structures of Chlide:LPOR:NADPH – additional information.

Helixplorer[6] symmetry determination plots and resolution estimation by gold-standard Fourier-shell correlation (GSFSC) of HF-23 (**A**), HF-25 (**B**), HF-29 (**C**), RF-21 (**D**), RF-23 (**E**), RF-25 (**F**) and SF-25 (**G**) structures as well as resolution estimation by gold-standard Fourier-shell correlation (GSFSC) of RD-21 (**H**), RD-23 (**I**), RD-25 (**J**) and RD-A (**K**). On the left: average power spectra (PS) in inverted gray-scale (stronger spots are black) for each type of helical assembly overlaid with the theoretical positions of Bessel function maxima. These maxima positions depend on the helical symmetry parameters used (pitch and axial rise). The parity of Bessel function orders are conditioned by the parity of additional cyclic/dihedral symmetry (Cn/Dn) that might also be present. Small blue and red dots on the right side of the PS indicate the expected positions of even and odd Bessel orders maxima, respectively. Insets show representative 2D class averages for each type of helical assembly. The intensity of spots in the PS depend both on the size, shape and orientation of the asymmetric unit and on electron microscope contrast transfer function (CTF). The CTF strongly attenuates low spatial frequencies near the PS center which explain why the pitch spots in (C) and the axial rise spots in (D,E,F) are not visible in the PS. On the right: Fourier shell correlation curves for the reconstructions presented in Figure 2 with different kinds of masks applied. **L.** 2D histograms of particle orientations (azimuth vs. elevation) for all the maps.

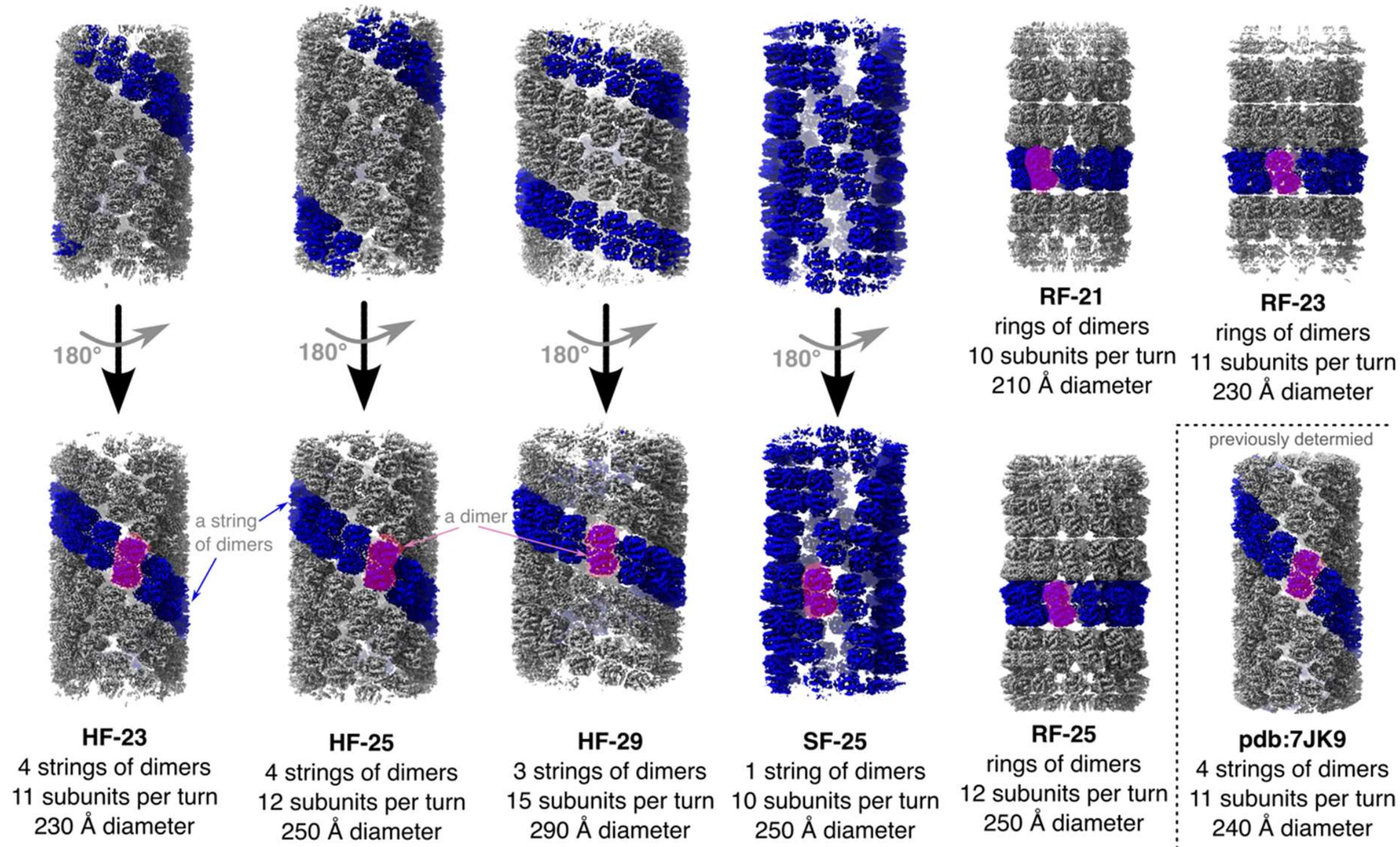

Supplementary Figure 3. Cryo-EM maps of the oligomeric assemblies of LPOR determined in this study comparing to the previously determined assembly. A single dimer is highlighted in pink, and one representative string of dimers is highlighted in blue. The number and arrangement of strings vary between assemblies, as indicated below each map.

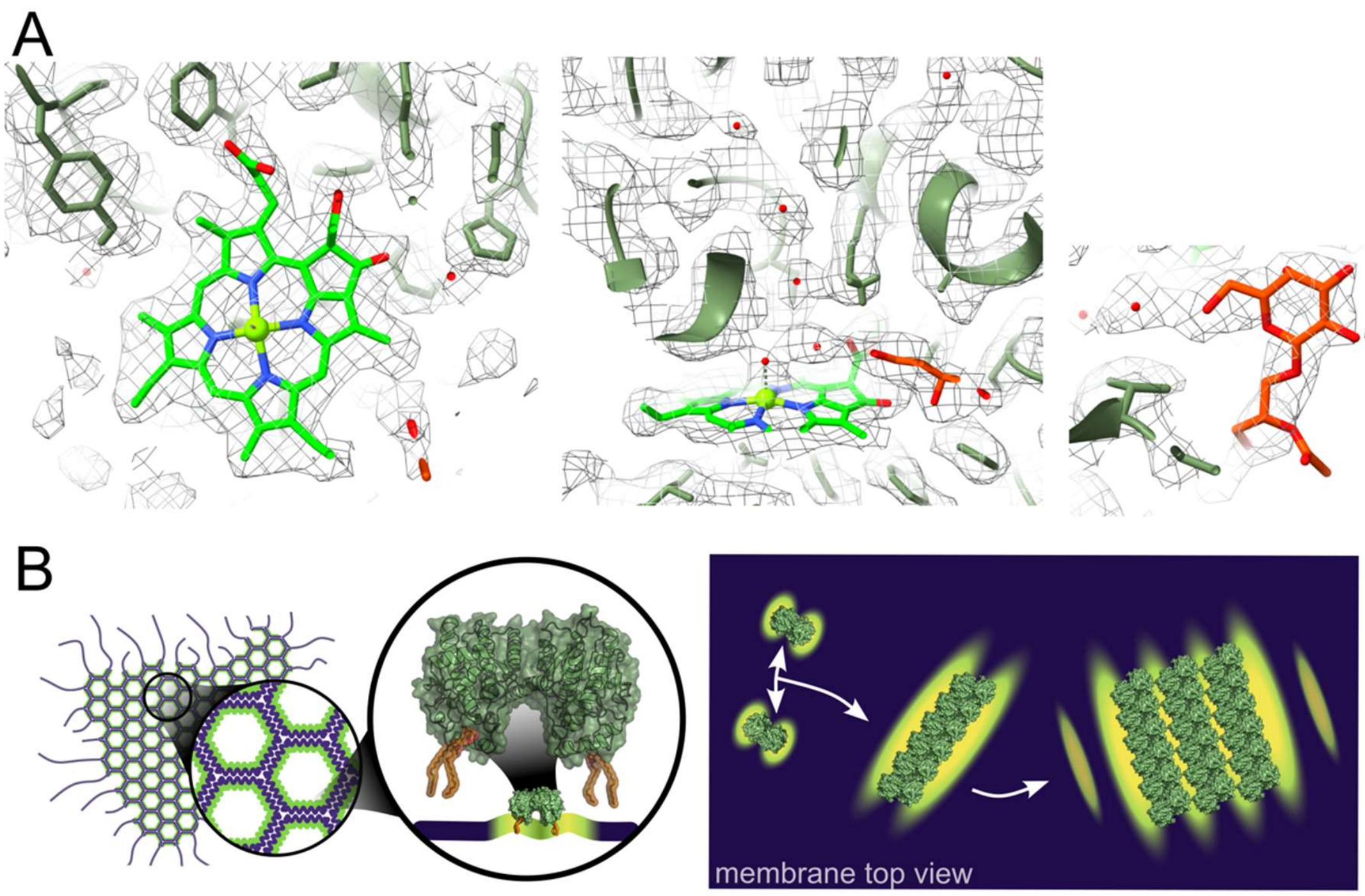

Supplementary Figure 4. MGDG-bidning site and LPOR interaction with the membrane.

A. Densities within pigment binding pocket, showing Chlide, water molecules an MGDG.

B. Schematic model of LPOR interaction with the membrane. Each dimer interacts with two MGDG molecules, inducing local membrane that promote further oligomerization. These periodic deformations are likely essential for stabilizing the ultrastructure of PLB.

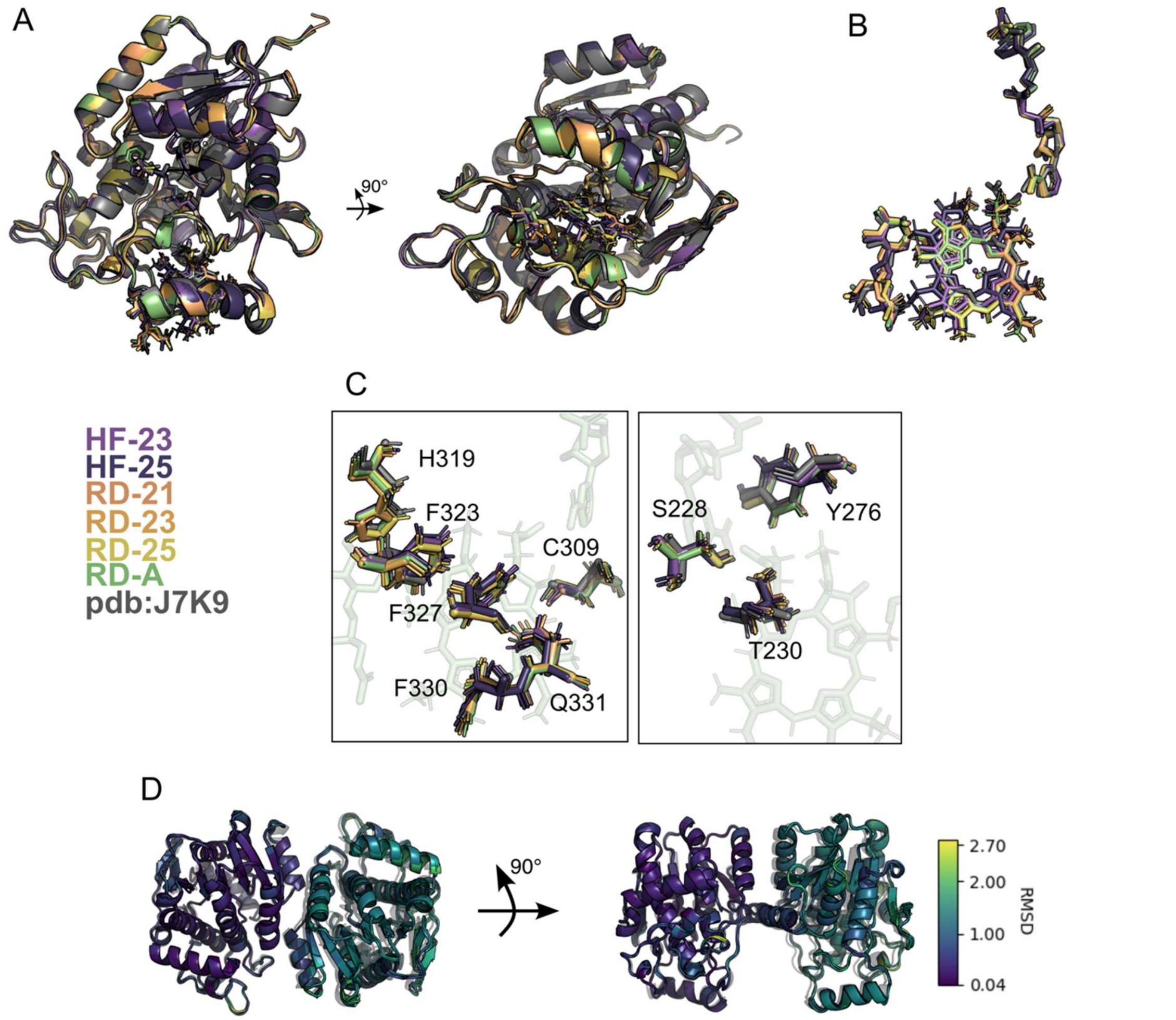

Supplementary Figure 5. LPOR dimers in all assemblies are essentially identical. **A.** Comparison of subunits from all assemblies, including the subunit of Pchlide:LPOR:NADPH complexs from pdb:7JK9**. B.** Overlay of NADPH, Pchlide and MGDG in LPOR subunit form all assemblies.

C. Conformation of selected residues involved in Pchlide/Chlide binding in subunits from all assemblies, including the subunit of Pchlide:LPOR:NADPH complexs from pdb:7JK9. **D.** Comparison of the conformation of LPOR dimers form all assemblies aligned to the dimer from PDB:7JK9 (in black semitransparent). The RMSD for each residue is shown in a color scale.

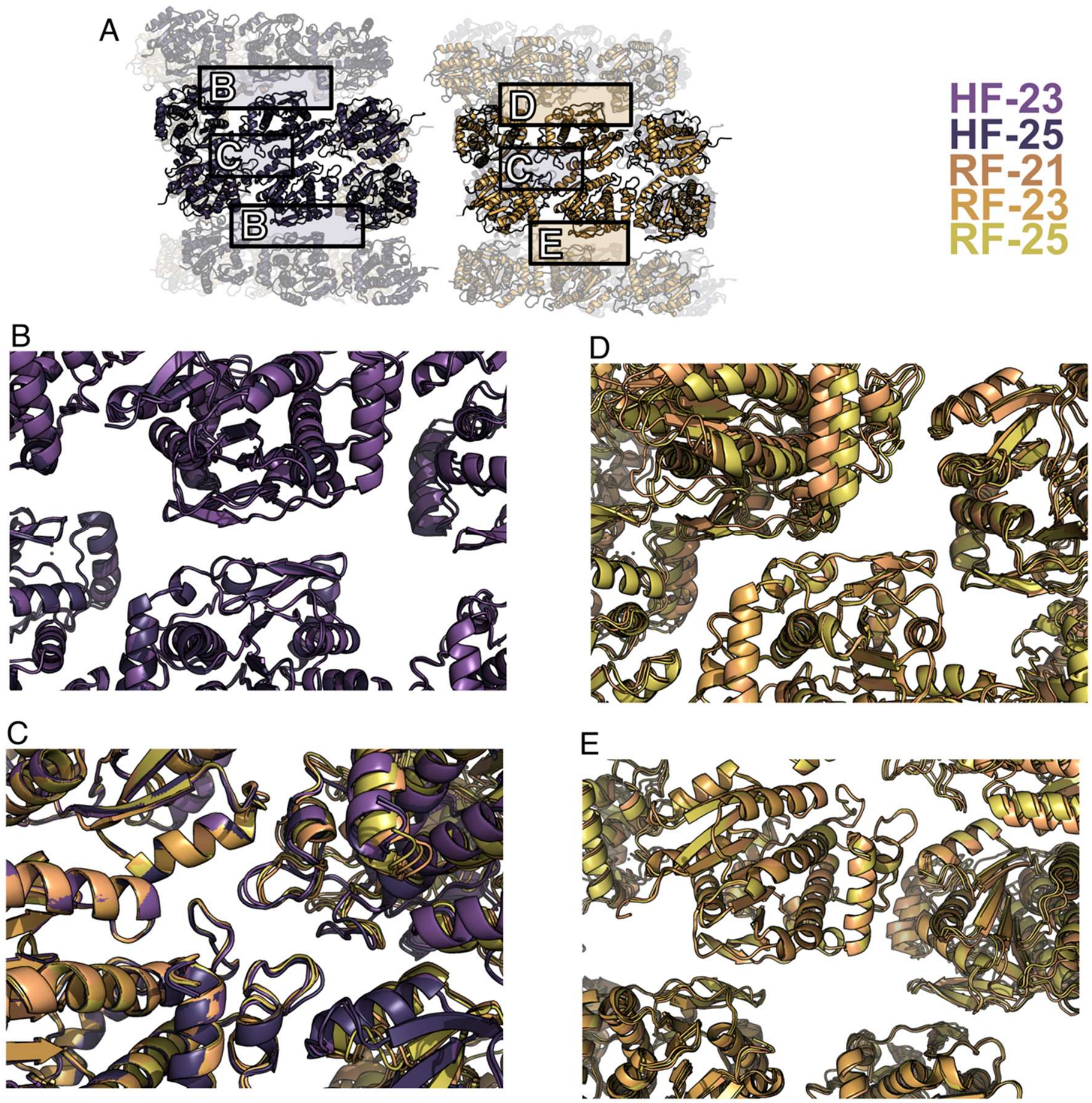

Supplementary Figure 6. Comparison of oligomerization interfaces between different assemblies.

A. A string of dimers in the helical (left) and stacked-ring (right) assemblies, with distinct oligomerization interfaces indicated.

DCDE. Close-up views of the oligomerization interfaces. Models are aligned based on a single dimer within the string.

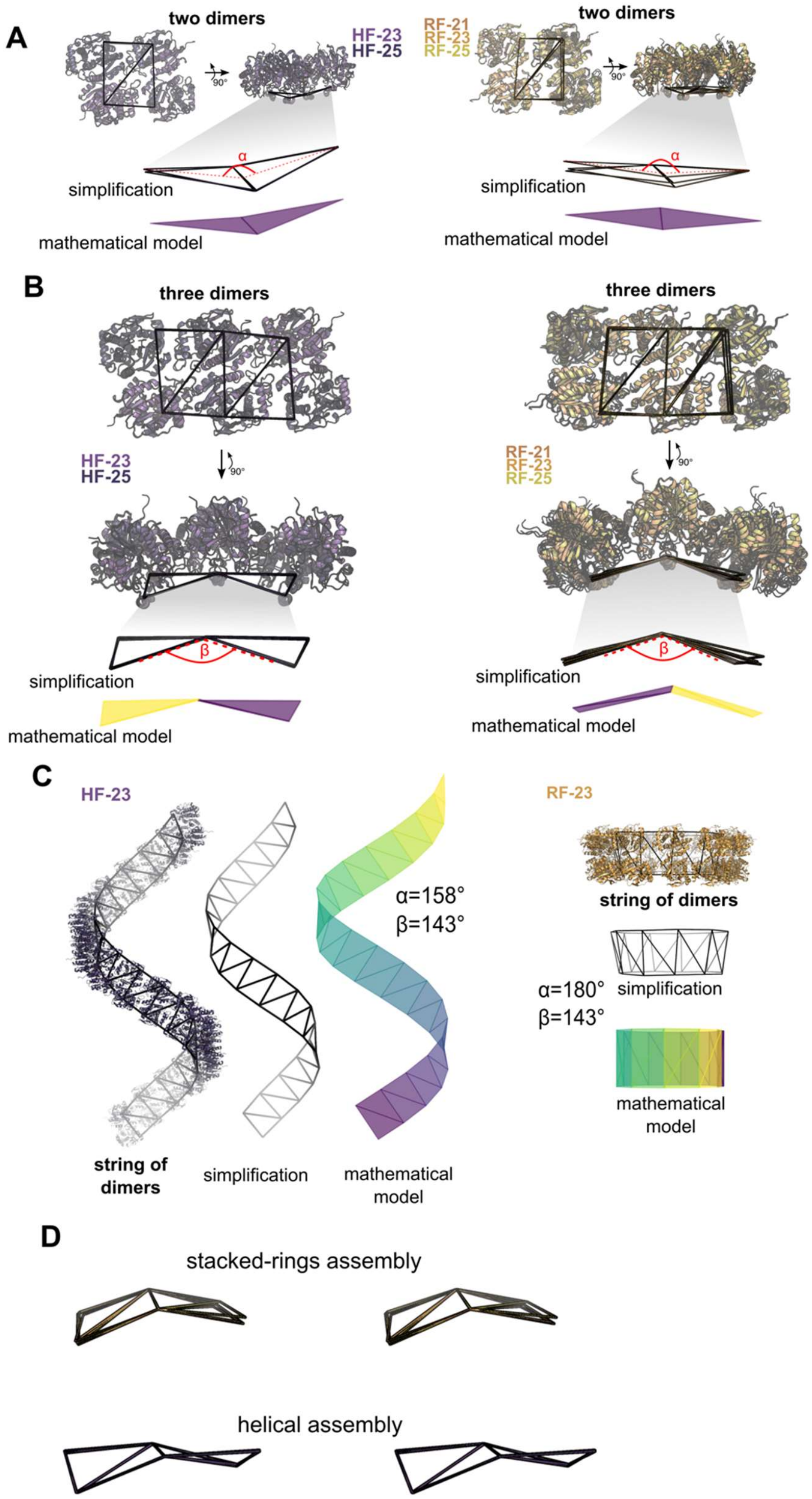

Supplementary Figure 7. Geometric principles of LPOR assemblies.
Each subunit within a string of dimers can be represented as a vertex of a square-based framework.
A. Two dimers form a square that is nearly planar in stacked-ring assemblies, whereas in helical assemblies the square is bent along its diagonal by an angle α.
B. Three dimers can be represented as two adjacent squares, with an angle β between the planes of the squares.
C. Both helical and stacked-ring assemblies can be described as strings of such squares, differing in the values of angles α and β.
D. Stereoscopic view of two adjacent squares in stacked-ring and helical assemblies.

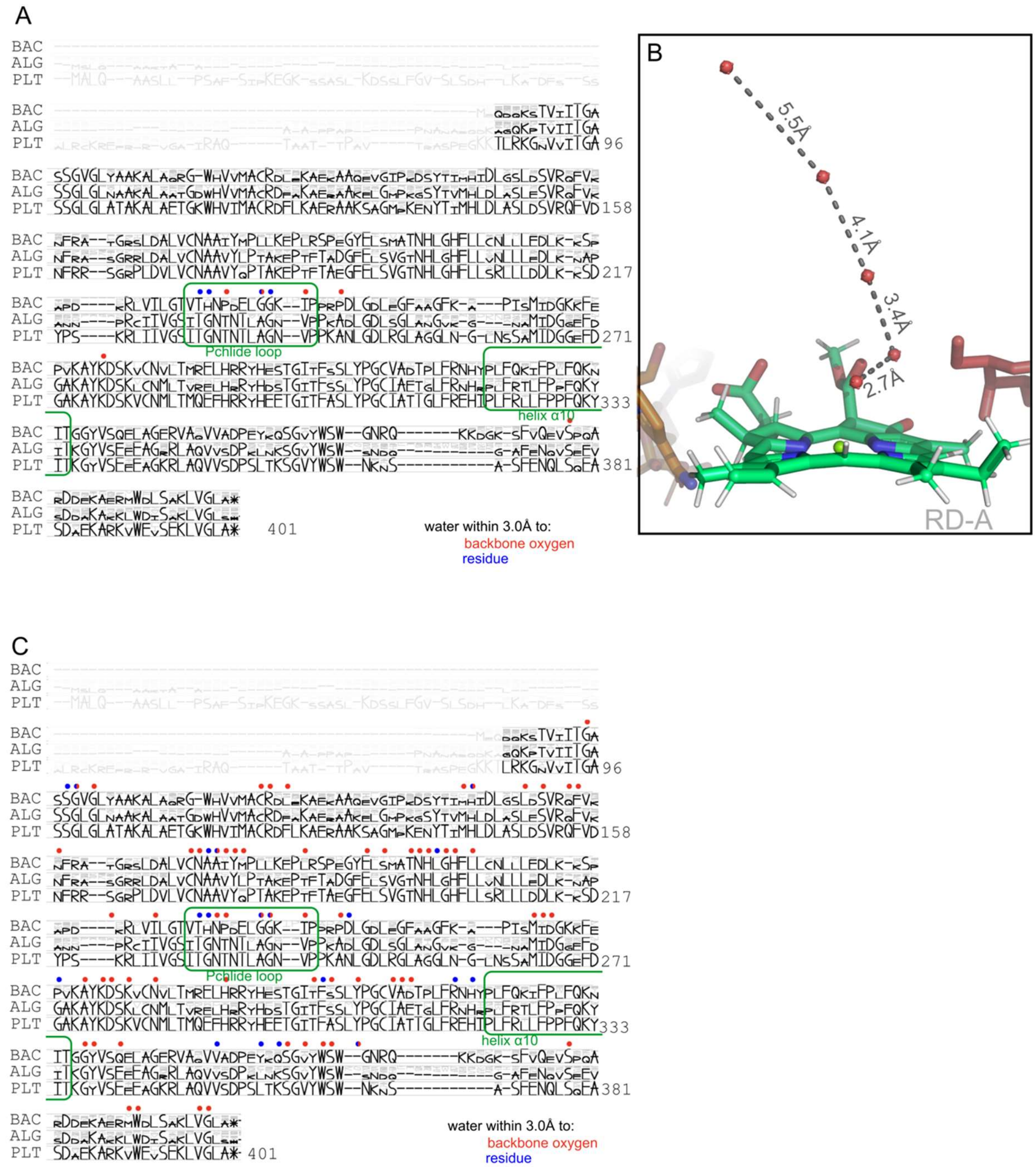

Supplementary Figure 8. Residues involved in water binding.

Sequences 346 of cyanobacterial (BAC), 19 algal (ALG), and 190 plant (PLT) aligned LPOR sequences. The size of each letter reflects the abondance of the given residue at a given position. **A.** Residues interacting with the water channel connecting the pigment with bulk solvent. **B.** The distances between the water molecules in the channel. **C.** The residues interacting with all water molecules present in RD-A model.

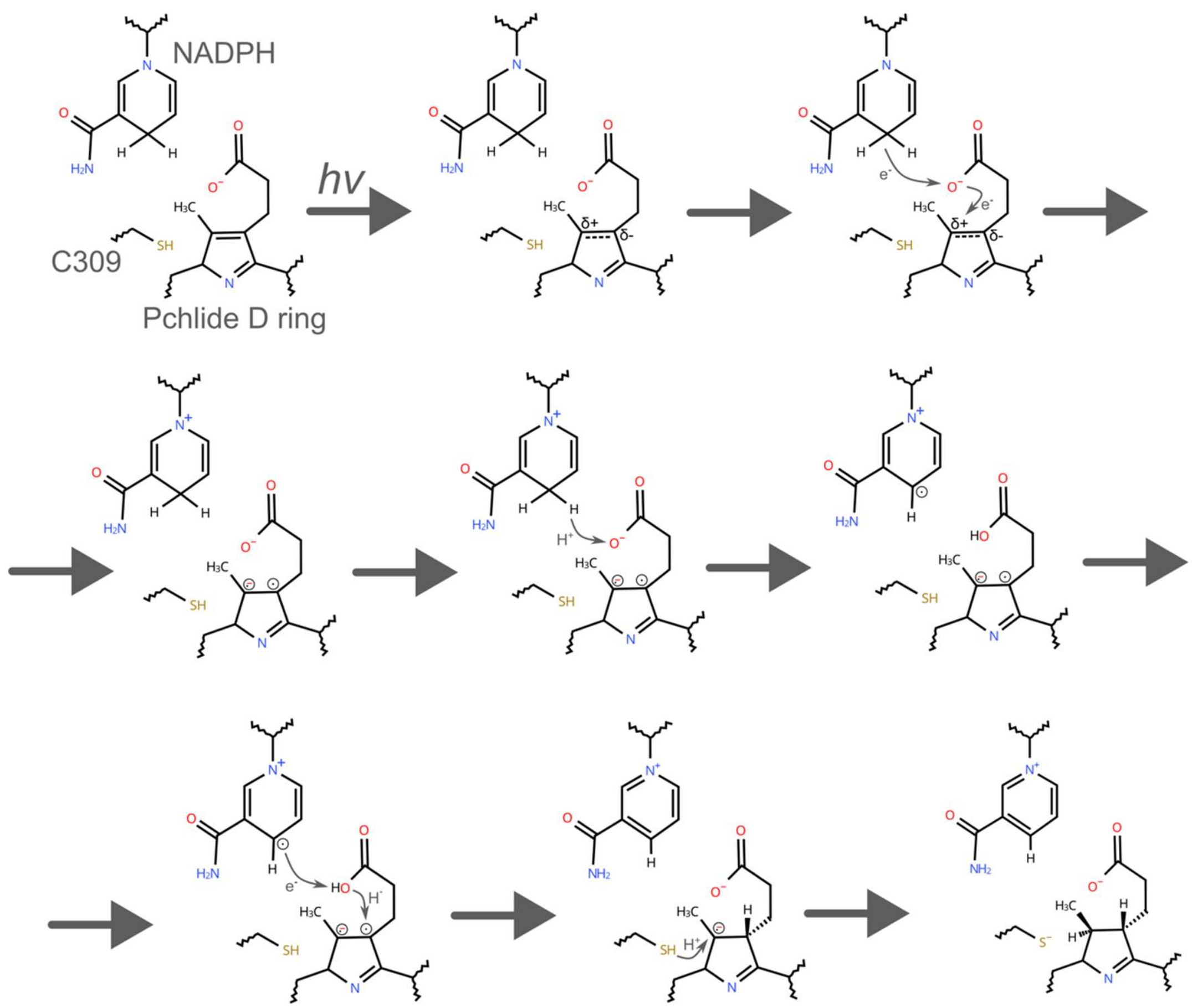

Supplementary Figure 9. Proposed novel reaction mechanism of Pchlide photoreduction catalyzed by LPOR.

Supplementary Table 1. RD-A cryo-EM data collection and refinement statistics.

Data collection	RD-A
Magnification	x105,000
Voltage (kV)	300
Electron Microscope	FEI Titan Krios G3i
Defocus (um)	-0.9 to -2.7
Pixel size (Å)	0.86
Total dose (e^-/ $Å^2$)	40.39
Number of frames	40
Number of micrographs	18 466
**Refinement**	
Number of total particles	1 340 624
GS-FSC Resolution (0.143, Å)[a]	2.55
Model composition	
Chains	2
Protein residues	634
r.m.s.d.	
Bond lengths (Å)	0.002 (0)
Bond angles (°)	0.563 (0)
**Validation**	
MolProbity score	1.14
Clash score	3.49
Ramachandran plot	
Favored (%)	98.57
Allowed (%)	1.43
Disallowed (%)	0.00
CC Mask	0.87

[a]Gold-Standard Fourier-Shell Correlation

Supplementary Table 2. RD-21 cryo-EM data collection and refinement statistics.

Data collection	RD-21
Magnification	x105,000
Voltage (kV)	300
Electron Microscope	FEI Titan Krios G3i
Defocus (um)	-0.9 to -2.7
Pixel size (Å)	0.86
Total dose (e^{-}/ Å^{2})	40.39
Number of frames	40
Number of micrographs	18 466
Refinement	
Number of total particles	380,180
GS-FSC Resolution (0.143, Å)[a]	2.64
Model composition	
Chains	2
Protein residues	638
r.m.s.d.	
Bond lengths (Å)	0.003 (0)
Bond angles (°)	0.757 (1)
Validation	
MolProbity score	1.55
Clash score	4.16
Ramachandran plot	
Favored (%)	97.63
Allowed (%)	2.37
Disallowed (%)	0.00
CC Mask	0.86

[a]Gold-Standard Fourier-Shell Correlation

Supplementary Table 3. RD-23 cryo-EM data collection and refinement statistics.

Data collection	RD-23
Magnification	x105,000
Voltage (kV)	300
Electron Microscope	FEI Titan Krios G3i
Defocus (um)	-0.9 to -2.7
Pixel size (Å)	0.86
Total dose (e^{-}/ $Å^{2}$)	40.39
Number of frames	40
Number of micrographs	18 466
**Refinement**	
Number of total particles	475,068
GS-FSC Resolution (0.143, Å)[a]	2.66
Model composition	
Chains	2
Protein residues	634
r.m.s.d.	
Bond lengths (Å)	0.003 (0)
Bond angles (°)	0.622 (0)
**Validation**	
MolProbity score	1.35
Clash score	2.89
Ramachandran plot	
Favored (%)	98.10
Allowed (%)	1.75
Disallowed (%)	0.16
CC Mask	0.82

[a]Gold-Standard Fourier-Shell Correlation

Supplementary Table 4. RD-25 cryo-EM data collection and refinement statistics.

Data collection	RD-25
Magnification	x105,000
Voltage (kV)	300
Electron Microscope	FEI Titan Krios G3i
Defocus (um)	-0.9 to -2.7
Pixel size (Å)	0.86
Total dose (e^-/ Å^2)	40.39
Number of frames	40
Number of micrographs	18 466
Refinement	
Number of total particles	485,376
GS-FSC Resolution (0.143, Å)[a]	2.58
Model composition	
Chains	2
Protein residues	636
r.m.s.d.	
Bond lengths (Å)	0.003 (0)
Bond angles (°)	0.646 (0)
Validation	
MolProbity score	1.52
Clash score	2.78
Ramachandran plot	
Favored (%)	97.31
Allowed (%)	2.53
Disallowed (%)	0.16
CC Mask	0.84

[a]Gold-Standard Fourier-Shell Correlation

Supplementary Table 5. RF-21 cryo-EM data collection and refinement statistics.

Data collection	RF-21
Magnification	x105,000
Voltage (kV)	300
Electron Microscope	FEI Titan Krios G3i
Defocus (um)	-0.9 to -2.7
Pixel size (Å)	0.86
Total dose (e^{-}/ $Å^{2}$)	40.39
Number of frames	40
Number of micrographs	18 466
**Refinement**	
Number of total particles	21,895
GS-FSC Resolution (0.143, Å)[a]	3.17
Model composition	
Chains	40
Protein residues	12,760
r.m.s.d.	
Bond lengths (Å)	0.003 (0)
Bond angles (°)	0.598 (0)
**Validation**	
MolProbity score	1.23
Clash score	4.61
Ramachandran plot	
Favored (%)	98.44
Allowed (%)	1.56
Disallowed (%)	0.00
CC Mask	0.81

[a]Gold-Standard Fourier-Shell Correlation

Supplementary Table 6. RF-23 cryo-EM data collection and refinement statistics.

Data collection	RF-23
Magnification	x105,000
Voltage (kV)	300
Electron Microscope	FEI Titan Krios G3i
Defocus (um)	-0.9 to -2.7
Pixel size (Å)	0.86
Total dose (e^-/ $Å^2$)	40.39
Number of frames	40
Number of micrographs	18 466
**Refinement**	
Number of total particles	25,485
GS-FSC Resolution (0.143, Å)[a]	3.20
Model composition	
Chains	44
Protein residues	13,948
r.m.s.d.	
Bond lengths (Å)	0.004 (0)
Bond angles (°)	0.615 (0)
**Validation**	
MolProbity score	1.57
Clash score	5.49
Ramachandran plot	
Favored (%)	96.01
Allowed (%)	3.94
Disallowed (%)	0.05
CC Mask	0.79

[a]Gold-Standard Fourier-Shell Correlation

Supplementary Table 7. RF-25 cryo-EM data collection and refinement statistics.

Data collection	RF-25
Magnification	x105,000
Voltage (kV)	300
Electron Microscope	FEI Titan Krios G3i
Defocus (um)	-0.9 to -2.7
Pixel size (Å)	0.86
Total dose (e^{-}/ $Å^2$)	40.39
Number of frames	40
Number of micrographs	18 466
**Refinement**	
Number of total particles	25,029
GS-FSC Resolution (0.143, Å)[a]	3.18
Model composition	
Chains	48
Protein residues	15,264
r.m.s.d.	
Bond lengths (Å)	0.003 (0)
Bond angles (°)	0.632 (0)
**Validation**	
MolProbity score	1.47
Clash score	5.50
Ramachandran plot	
Favored (%)	96.97
Allowed (%)	2.94
Disallowed (%)	0.09
CC Mask	0.79

[a]Gold-Standard Fourier-Shell Correlation

Supplementary Table 8. HF-23 cryo-EM data collection and refinement statistics.

Data collection	HF-23
Magnification	x105,000
Voltage (kV)	300
Electron Microscope	FEI Titan Krios G3i
Defocus (um)	-0.9 to -2.7
Pixel size (Å)	0.86
Total dose (e^-/ Å^2)	40.39
Number of frames	40
Number of micrographs	18 466
Refinement	
Number of total particles	70,789
GS-FSC Resolution (0.143, Å)[a]	2.91
Model composition	
Chains	44
Protein residues	13,994
r.m.s.d.	
Bond lengths (Å)	0.004 (0)
Bond angles (°)	0.536 (173)
Validation	
MolProbity score	1.14
Clash score	2.56
Ramachandran plot	
Favored (%)	97.50
Allowed (%)	2.50
Disallowed (%)	0.00
CC Mask	0.89

[a]Gold-Standard Fourier-Shell Correlation

Supplementary Table 9. HF-25 cryo-EM data collection and refinement statistics.

Data collection	HF-25
Magnification	x105,000
Voltage (kV)	300
Electron Microscope	FEI Titan Krios G3i
Defocus (um)	-0.9 to -2.7
Pixel size (Å)	0.86
Total dose (e^-/ $Å^2$)	40.39
Number of frames	40
Number of micrographs	18 466
Refinement	
Number of total particles	23,139
GS-FSC Resolution (0.143, Å)[a]	3.08
Model composition	
Chains	48
Protein residues	15,120
r.m.s.d.	
Bond lengths (Å)	0.003 (0)
Bond angles (°)	0.559 (38)
Validation	
MolProbity score	1.30
Clash score	3.91
Ramachandran plot	
Favored (%)	97.40
Allowed (%)	2.60
Disallowed (%)	0.00
CC Mask	0.87

[a]Gold-Standard Fourier-Shell Correlation

Supplementary References

1. Nguyen, H. C., Melo, A. A., Kruk, J., Frost, A. & Gabruk, M. Photocatalytic LPOR forms helical lattices that shape membranes for chlorophyll synthesis. *Nat. Plants* **7**, 437–444 (2021).
2. Kruk, J. & Myśliwa-Kurdziel, B. Separation of monovinyl and divinyl protochlorophyllides using C 30 reverse phase high performance liquid chromatography column: Analytical and preparative applications. *Chromatographia* **60**, (2004).
3. Gabruk, M., Myśliwa-Kurdziel, B. & Kruk, J. MGDG, PG and SQDG regulate the activity of light-dependent protochlorophyllide oxidoreductase. *Biochemical Journal* BCJ20170047 (2017).
4. Nowak, P., Michalik, M., Fiedor, L., Woźniakiewicz, M. & Kościelniak, P. Capillary electrophoresis as a tool for a cost-effective assessment of the activity of plant membrane enzyme chlorophyllase. *Electrophoresis* **34**, 3341–3344 (2013).
5. Fiedor, L., Rosenbach-Belkin, V. & Scherz, A. The Stereospecific Interaction between Chlorophylls and Chlorophyllase POSSIBLE IMPLICATION FOR CHLOROPHYLL BIOSYNTHESIS AND DEGRADATION. *J. Biol. Chem.* **267**, 22043–22047 (1992).
6. Estrozi, L.F., Desfosses, A., Schoehn, G., 2018. Helixplorer: Online Indexation of Fibers And Helical Structures, http://rico.ibs.fr/helixplorer/
