# Supplementary material for: Structures of LPOR–Chlide complexes reveal the structural basis of membrane remodeling and photocatalysis
Source: Nat Commun. 2026 Aug 5;17:9427. doi: 10.1038/s41467-026-76316-7 (PMC13538550; doi:10.1038/s41467-026-76316-7)
Supplement: Supplementary file 2 [file 41467_2026_76316_MOESM2_ESM.pdf]

Description of Additional Supplementary Files

File name: Supplementary Data 1
Description: The compressed archive contains the raw data and scripts used to generate Fig. 1 and. Fig. 5E.

File name: Supplementary Movie 1
Description: Overview of the maps determined in this study.
