# Supplementary material for: Structures of LPOR–Chlide complexes reveal the structural basis of membrane remodeling and photocatalysis
Source: Nat Commun. 2026 Aug 5;17:9427. doi: 10.1038/s41467-026-76316-7 (PMC13538550; doi:10.1038/s41467-026-76316-7)
Supplement: Supplementary file 4 [file 41467_2026_76316_MOESM6_ESM.pdf]

nature portfolio

Peer Review File

Structures of LPOR–Chlide complexes reveal the structural basis of membrane remodeling and photocatalysis

Corresponding Author: Dr Michal Gabruk

This file contains all reviewer reports in order by version, followed by all author rebuttals in order by version.

Version 0:

Reviewer comments:

Reviewer #1

(Remarks to the Author)
The manuscript Structures of LPOR-Chlide complexes reveal the structural basis of membrane remodeling and photocatalysis presents interesting structural results describing several different LPOR filament architectures from plants using single-particle cryo-EM. Unlike previous studies, this work focuses on Chlide-LPOR in complex with NADPH. The authors show that the LPOR dimer serves as the fundamental building block of the filaments and analyze the periodic deformation of lipid bilayers associated with different filament architectures. Furthermore, based on the high-resolution structures, the authors propose a reaction mechanism consistent with their structural observations. The cryo-EM data appear to be of good quality and are potentially important.
However, at present, the structural novelty of this work are not sufficiently clearly defined. In particular, the manuscript does not adequately distinguish the newly identified filament architectures from those previously reported, including earlier work by the same authors. A clearer comparison with prior studies is essential to establish what is genuinely structurally new and what confirms or extends existing models.
In addition, the proposed reaction mechanism, while plausible, is not experimentally validated in this study. Therefore, either additional supporting evidence should be provided, or the mechanistic interpretation should be presented more cautiously as a proposed model rather than a definitive mechanism.
Overall, the work has the potential to make an important contribution, but its novelty and mechanistic conclusions need to be more clearly articulated. With improved clarification the significance of the findings would be easier to assess.

Major points:
The authors must show a 3D model (e.g. schematically) in the introduction (Either main figure or supplement) where the reader can properly see the current model of the PLB/cubic phase 3D structure of the etioplast. It is very hard to imagine this from the text only. It is known from reference 9 and reference 10 how these organize. It should be shown schematically and it should be specially shown how the LPOR is involved in the organization of the 3D model of the cubic phase/prolamellar bodies. It is unclear for the reader how the proteins are involved in this formation especially because the lipids are trapped into the filament. How is this incorporated into a 3D PLB? This model should then later reappear in the last figures and it should be adapted by the structures found in this paper. Which filaments were known what was not known etc. also how do the filaments described in this paper relate to the filament described before in that model? Then it is also better to judge what exactly is the novelty of this study compared to what was published before.
Fig 1F: There needs to be a control micrograph. Here it only shows after 10 min the filament is stable but where is the reference micrograph where it would show that with low NADPH concentration it should disassemble or partially disassemble.
The section and Fig 2D of LPOR assembles into a variety of oligomeric states must be much better described in terms of protein chain composition. How many protein chains form each different filaments what is the packing how does it relate to the filament thickness etc. For example the dimer could be highlighted in the helical filaments then it should show how many dimers in a full rotation or rise twist and pitch directly labelled on each filament. Equally for the ring filaments, as it is already described in the text how many dimers are forming it, it should show it for example by rotating 90 degrees to look down the axis and then to count the number. In Line 211 onwards It says 4 and 3 and 1 strings of dimers. What does it mean? Please highlight those in the figure and label. Also a comparison with previously determined filaments of Pchlide should be shown in supplement.
Fig 4 ABC: These panels are very hard to understand especially for the definition of alpha and beta angle. These angles need to be better shown in context of model. Where exactly are the alpha and beta in the model Also the many squares

shown with many lines are unclear and confusing. Also it is unclear why only the model with this interaction in panel E F G H are shown only for HF23 and RF23. Any justification for that? In line 327, 335, and 329 it says mirror-symmetric interaction/arrangements. This is very misleading because there are no mirror symmetries possible in proteins. This whole chapter is very difficult to understand and should be simplified substantially.
Extended data Fig 5 should not claim novel reaction mechanism because it is not confirmed it is rather proposed. Also it would be great to see the mechanism that was proposed previously to this study side by side as comparison and why the old mechanism would fail based on the structure. This mechanism should be confirmed by some independent method or very downweighted in claim and said this is a proposed alternative mechanism.

Minor points:
Line 45: It says most models adopt helical or stacked ring forms whereas one forms a string of dimers not seen before. It is unclear to the reader if this "not seen before" is referring only to LPOR or in general. Does this mean the other filaments were seen before in other cases (not LPOR)? This needs to be properly written, what exactly is new and what exactly was not seen before.
Line 51: Again confusing with what was written before. It said one string dimer in line 46. Now it says about several different string architectures. Is it now one or many different structures? Again this must be much better phrased and explained so that the readers are not confused.
Line 66: Should mention that LPOR + Protochlorophyllide + NADPH + light ⇒ Chlorophyllide with references
Line 67: No reference for LPOR involved in cubic phase remodelling
Line 74: "whose main lipid component is the non-bilayer-forming monogalactosyldiacylglycerol (MGDG)" MGDG are forming bilayers in those paracrystalline structures or not? If they do then it would be better to remove non-bilayer forming because it suggests they are not forming bilayers in the cubic phase but they likely do. This non-bilayer forming refers to isolated MGDG.
Line 76: "Thus far, a few crystal structures of cyanobacteria …" Should be changed to Several crystal structures …
Line 78: However, the cryo-EM structure of the … : remove However because it was used in the sentence before.
Line 79: Should be revealed the binding pocket with its substrate bound
Line 85: redshifts the pigment from ? to 655 nm at 77K …
Line 90: double bond 17–18 for the D ring of the tetrapyrrole
Line 98: Please rephrase to "Immediately after the product (Chlide) formation" for clarity
Line 104: rephrase: which is associated with subsequent esterification of Chlide by chlorophyll synthase, that completes Chl synthesis.
Line 105: … what ultimately leads to PLB…. Should be … which ultimately leads to PLB
Line 122 : Fig1A: I do not see the shaded areas which represent the standard deviation
From Line 120 onwards: Here it describes the monitoring of the fluorescence spectra temperature dependent. In the methods it is however not clear how this is performed. First seedling is grown in dark. Then measurements occur one by one I suppose. First 80K, illumination, recording, then next temperature from the same sample, illumination recording etc. It says namely: For each target temperature after the initial 80 K measurement (without prior illumination), the sample was illuminated for 1 min with saturating white LED light (> 6000 μmol photons m^{-2} s^{-1}) and then dark-incubated for 1 min at the target temperature.
Is the phrase without prior illumination now referring to 80K point? It is confusing. Is it first illuminated at 80K then measured and then equilibrated to the next temperature then illuminated again, then 1 min dark, then measured? Or is it illuminated only once? The authors should write that in a more precise way.
Line128: Chlide or Pchlide?
Line 134 use NADPH instead of dinucleotide throughout the manuscript to be consistent
Line168 Fig 1E not F
Line 171 Fig 1F not E
Line 177 It says to elucidate how LPOR accommodates both Pchlide and Chlide we perform helical reconstruction of Chlide LPOR NADPH OPT but what about the Pchlide? Rephrase for example remove Pchlide if in this study you did not investigate it.
Line 201 18,466 movies not 18466 videos
Extended Fig 1C: Please mention if this is in vitro reconstituted sample in the caption. What does it mean complexes of different composition, clarify in detail.
Figure 1B: It is not clear what those curves mean since there is no explanation in the caption of the color code etc. Also shaded areas I cannot identify. It needs to be explained better what this panel exactly means. Also there is this Shibata shift twice depicted. In vivo and in vivo. Is it meant to be in vivo and in vitro please clarify.
Fig 1C: In the text it says the shift is composed of two different components. In the figure 1C please mark the components. Is it meant that the emission spectra shifts from 690 nm to 681 nm as the 2 components. If yes then better not use the word components because it suggests that it is somehow a sum of two components. Please rephrase.
Extended Data Fig 1AB: what is the difference between the two? Simply replicates? In any case mention what is what exactly.
Extended data figure 2: Please provide all angular distributions.
Fig 1D. This is unclear diagram: It needs explanation. The NADPH is used in the reaction and NADP+ will be produced. Does NADP+ bind or is it unable to bind please clarify. Then all of a sudden NADPH drops during Shibata shift. No explanation is given why. Is there any process that uses NADPH?
Fig. 2 D: It is hard to understand what these + signs mean. Please clarify in detail in the caption what was done. I suppose the RF-21 RF23 and RF25 were averaged. To obtain RDA. If so please give justification to do this in the text and also make clear in the caption that the RF-21 RF23 and RF25 were averaged. Are they really the same to allow averaging? Also add particle number for each subset
Extended Data Table 3, 5, 6, 7: Model validation is insufficient in terms of Ramachandran outliers and allowed region. The

expectation value is 98, 2, 0. For example 93% with 5.75% allowed is energetically unfavorable. Please fix this.
Line 223: Confusion. You show 9 maps 7 filaments and then it says we have 11 maps. Please clarify. Either only mention 9 or say specifically each map and why are the others not shown in Fig 2?
Fig 3D please show density as primary proof for lipid and water and pigment
Fig 3A please label outer and inner leaflet
Fig 3 E and F should be one panel? In the caption the E is missing. In the text as well the panel E is not called. Also the panel is way too small. Maybe move into supplement and make it much larger and label it properly so that the reader can actually see what is shown.
Line 311 This subtitle is not very informative. Please change more towards a combination of three different interaction modes between dimers defines the higher oligomeric string formations of filaments
Line 314: It says: two dimers can interact at least in two distinct ways …. (Fig 4A). In Fig 4A however it does not show two distinct ways. At least it is hard to see from that. Please improve this.
Line 316: The square with a subunit in each corner. Please clarify where the corner is placed in the text? It seems from the figure it is placed always in different position of the subunit. Should it not be placed every time in the same exact position? If not define the position of the corner properly and justify the choices.
Line 315: …the square is nearly planar … It is not clear what this means please rephrase and explain in detail what it means
Fig 4 panel B This panel should show the interactions. But it does not show any dashed lines that would point to this. Also it is better to make two panels side by side with RF23 and indicate the differences if any. Please remove hydrogens they make the figure hard to understand
Fig 4 panel E G H, again no interactions are shown. Please show the interactions with dashed lines and also remove the hydrogens. What are these transparent models in the background. Remove because they are also very confusing.
Fig.5: Again please remove hydrogens they are not very useful. Also it is not consistent because there are no hydrogens shown for water. In addition sometimes they are even in the wrong orientation. For example T230 forms a hydrogen bond with ligand but the hydrogen points opposite direction.
Fig 5 D the C17–C18 bond is not planar this is misleading please show it with planarity
Line 372 2x).)., remove one
Line 457: FLU should be written the FLU (FLUORESCENT protein)
Line 472: VIPP1 should be explained what it is in 1–2 sentences.
Line 437: In the results part it is said in Line160 that it resembles the Chlide:LPOR spectra without NADPH bound. Here it says Chlide is released. Please clarify what is released in which order. It is confusing. Is not the release of NADPH responsible for the Shibata shift? Please clarify.
Line 481: Please change: A Novel Reaction Mechanism Consistent with Previous Observations to something more like Proposed mechanism for the …
The whole paragraph is written as if the mechanism is kind of figured out, and now only we need to study some more details. However this is not the case, as suggested before either experimental verification and validation need to be provided or the whole paragraph should be written more speculatively and downweighted.
Line 541 to 543: Although referencing the old studies is good also to spell it out in shorter form what was done here. This is true for all references in the methods. Please briefly describe what was done not just referencing back.
Line 560: All scripts in this manuscript must be provided in the supplement material
Line 562: Again please describe what was done not just reference back
Line 569: Please clarify why such high amount of Arginine is needed. Could it lead to artifacts in the filament formation?
Line 585 18466 should be 18,466
Line 591: Mention the word “total dose” which is 40 $e^-/Å^2$

Reviewer #2

(Remarks to the Author)
In this mauscript, Gabruk et al. revealed the molecular structures of LPOR-Chlide-NADPH complexes associated with the lipid membrane. Their findings provide novel insights into the reaction mechanism of LPOR, and surprisingly suggest the limited structural changes in LPOR after the photoconversion of Pchlide. These observations lead us to reconsider current models of the LPOR reaction and subsequent remodeling of PLB.
Although this manuscript includes intriguing data and arguments, I am concerned that some of the authors’ interpretations may go beyond the data presented, particularly in the following two aspects: (1) The relationship between the LPOR structure and PLB disassembly, and (2) how the structure identified here relates to LPOR function in mature chloroplasts rather than in etioplasts (or etio-chloroplasts). I strongly recommend that the authors reconsider these points to make their manuscript more coherent and convincing.

Major points

1. Lines 119 and 419 and their related sections. I think the authors’ data are insufficient to conclude that Chlide:LPOR:NADPH complex formation or NADPH concentration affects the morphological changes of PLBs. I understand that spectral changes in vivo coincide with PLB dissociation and the persistence of LPOR oligomers after light exposure, however, the current data do not indicate in which conditions the PLB structure was disassembled. To clarify this causal relationship, it would be beneficial to perform in vitro experiments using different NADPH concentrations. Moderating the interpretation may be another option. The title and the last sentence of the abstract should also be reconsidered in this regard.

2. Lines 238, 266-271 and 464-479. The authors claim that LPOR oligomers affect the membrane structure due to their interaction with MGDG. This may be true, however, doesn’t the same apply to LPOR oligomers with Pchlide, given that the

ternary complex structures are largely unchanged before and after photoconversion of Pchlide? I think that that the alternation of membrane structure cannot be explained solely by the interaction between LPOR and MGDG, but may also involve additional proteins like CURT1 or other lipids like SQDG or PG, which have charged head groups. As the Extended Figure 3 showing the similarity between Pchlide:LPOR;NADPH and Chlide:LPOR:NADPH is very important, I recommend that the authors include it in the main figure.

3. Throughout the manuscript, the discussion related to PLB formation, PLB disassembly, and chlorophyll biosynthesis in mature chloroplasts appears to be mixed. For instance, lines 274-283 discuss the PLB formation based on data from Chlide:LPOR:NADPH complexes. The effects of photosynthesis discussed in lines 446-455 cannot be directly applied to understand the process in etioplasts or the very beginning phase of etioplast-chloroplast differentiation. Also, LPOR oligomers and PLBs are specific to etioplasts, thus regulatory mechanism of LPOR in mature leaves (lines 462 and 503) may be largely different from those observed in dark conditions. Please reconsider if the current data truly support the proposed mechanism of PLB formation or chlorophyll biosynthesis under continuous light conditions (without PLB).

Minor points

1. Line 67. Please clarify the evidence that the cubic phase is required for chloroplast development.

2. Figure 1 B and C. Please explain what the different colors indicate. In Fig. 1C, I failed to understand the meaning of x-axis. Please clarify.

3. Figure 2. Do the authors have any suggestions about which size of LPOR filaments are similar to those observed in etioplasts?

4. Lines 177-179 and 282-283. Explaining the purpose of next experiment at the end of sections is somewhat unusual. It would be more straightforward to put these sentences at the beginning of the next section.

5. Line 513. Triticum aestivum should be written in italics.

I hope my comments are helpful for the authors.

Reviewer #3

(Remarks to the Author)
The article by Gabruk and co-workers, entitled 'Structures of LPOR–Chlide complexes reveal the structural basis of membrane remodeling and photocatalysis' presents new cryo-EM structures of complexes of the light-dependent enzyme LPOR in complex with the product of the photoreaction Chlide. The LPOR enzyme is crucial for both chlorophyll biosynthesis and chloroplast development but crucial structural information on the substrate and product complexes has been lacking. This paper builds on previous cryo-EM work by the same authors where they presented structures of the LPOR-Pchlide (i.e. substrate) complex. Here, the improved resolution of the LPOR-Chlide complexes has provided important information on how these complexes shape the photosynthetic membranes, as well as new insights into the catalytic mechanism of the enzyme. The novelty of the work definitely arises from the higher resolution structural data provided by the elegant cryo-EM data, which appear to be very well carried out. However, some of the interpretations discussed are not completely new and the authors seem to have omitted quite a singificant chunk of older and newer literature. Hence, I have a few concerns that need to be addressed prior to publication:

1. The data and findings presented in figure 1 are not new and the molecular events following photocatalysis have been known for more than 30 years. Some of the older literature can be found in a review by Lebedev and Timko in 1998 (DOI: 10.1023/A:1006082119102) but studies by Boddi and co-workers on PLBs (e.g. Boddi, B., Ryberg, M., and Sundqvist, C.,1993 Analysis of the 77K fluorescence emission and excitation-spectra of isolated etioplast inner membranes, J. Photochem. Photobiol. BsBiol. 21, 125-133; Boddi, B., and Franck, F., 1997 Room-temperature fluorescence spectra of protochlorophyllide and chlorophyllide forms in etiolated bean leaves, J. Photochem. Photobiol. BsBiol. 41, 73-82) show very similar results to those presented here. In addition, the sequence of product release and co-enzyme rebinding steps have also been shown in vitro, albeit on cyanobacterial enzymes by Heyes and co-workers (DOI: 10.1021/bi049576h; https://doi-org.manchester.idm.oclc.org/10.1074/jbc.M706098200). In the latter, they even present rate constants for each of these steps. The authors need to state what additional data they have found in their work and how it is significantly different to this previous work. They also need to reference all of this previous work and place their work in the context of these previous findings. In my opinion the novelty of these initial results presented in Fig 1 is the fact that Chlide-LPOR-NADPH still forms large oligomeric complexes (Fig 1E). Do they have similar TEM data in the presence of excess NADP+ for the Chlide-LPOR-NADP+ complexes or in the absence of coenzyme (Chlide-LPOR)?

2. For the cryo-EM work the authors add 40uM Chlide to 28uM enzyme (i.e. close to 1:1). It would seem to me that this would result in a significant proportion of unbound enzyme or just with NADPH bound to the enzyme. Do the authors see any such complexes and how can they be sure that the enzyme is fully bound? Could they also comment on the order in which they mix the components together and does this make a difference? For example what happens if you add the NADPH or the lipids last?

3. The conformation of the LPOR-Chlide-NADPH dimers is essentially the same as the previously determined LPOR-Pchlide-NADP+ complexes but can they discuss how these compare to those described for the cyanbacterial system also

determined by cryo-EM (https://doi-org.manchester.idm.oclc.org/10.1111/febs.15542). There is no mention of any comparison to this other previous work.

4. In extended data figure 3C the authors show a comparison with their previous structures and highlight 3 Phe residues which appear to be very close together. Do these interact with the pigment in any way? Can the author comment on the importance of these residues as it has been shown that some of these Phe residues are important for catalysis (https://doi-org.manchester.idm.oclc.org/10.1111/febs.15542).

5. One of the key novelties of the paper that results from the higher resolution of the EM data is the protruding propionate group at the C17 position of the pigment molecule and the implications of this for the catalytic mechanism. In the mechanism shown in Fig 5D and Extended data figure 5 they show that after an initial electron transfer from NADPH there is then a proton transfer followed by another electron transfer (both from NADPH). Previous computational studies from Johannissen et al (ref 13) show that this is actually a H-atom transfer rather than 2 separate steps so the authors should change this accordingly.

6. Following on from the above point then I don't think it is correct for the authors to state that this reaction mechanism has not been examined directly before. They now have structural validation of the mechanism presented by Johannissen et al. This work also backs up the work shown by Taylor et al in 2023 (oi:10.1111/febs.17025) where a similar mechanism is proposed. In that paper they also point towards the importance of the Cys residue and the fact that the Tyr is key for pigment binding. The current work should be presented in terms of these latest, more recent findings rather than some of the older mechanistic literature which has since been revised.

Version 1:

Reviewer comments:

Reviewer #1

(Remarks to the Author)
The manuscript has been improved, and the comments have been adequately addressed. Extended Data Figure 3 is a very nice addition and significantly enhances the clarity of the presentation. I have no further comments.

Reviewer #2

(Remarks to the Author)
The authors intensively revised the manuscript and substantially improved it. However, two major concerns remain to be addressed.
1. Relationship between Chlide:LPOR:NADPH complexes and PLB disassembly. I understand that the concentration of NADPH changes the behavior of LPOR complexes after illumination. Nevertheless, this does not directly support the authors' idea that formation of Chlide:LPOR:NADPH complexes causes slow PLB disassembly. I agree with the description on page 18, lines 23-25: "Then NADPH rebinds in the LPOR binding pocket, stabilizing an LPOR oligomers with Chlide and transiently delaying PLB disassembly." However, I disagree with the phrases on page 2, lines 29-30, and page 5, line 2, because how the complex affects PLB disassembly is not clarified in the manuscript. Thus, I propose to modify the phrase in the abstract to "our data highlight the close relationship between Chlide:LPOR:NADPH complexes and structural changes of prolamellar bodies." The results shown in the manuscript indicates the importance of NADPH concentration in Chlide:LPOR:NAPDH oligomerization, but its consequences are not yet clarified. A previous study also demonstrated the role of NADPH in delaying PLB disassembly and spectral shift of Chlide, but neither of them proved a causal relationship between Chlide:LPOR:NAPDH complex formation and slow PLB disassembly. I do not object to suggesting such a relationship, but the current wording in the manuscript still sounds misleading for me.
2. Behavior of LPOR in developed chloroplasts. The authors' idea about LPOR oligomerization in chloroplasts lacks sufficient evidence. It is of course possible, and I think dimerization is very likely in chloroplasts due to its structural features, but there are no data demonstrating oligomer formation under prolonged light conditions. The Chlide:LPOR:NADPH complexes CAN be formed in chloroplasts, but this remains hypothetical. For such an argument, oligomerization of LPOR in mature leaves should be examined experimentally. In this regard, I strongly recommend that the authors modify (or remove) the following parts: page 2, lines 30-31; page 4, line 22; and page19, lines 2-22.

Minor point
Page 3, line 11. "The inner membrane" is expected to be rephrased as "the internal membrane", because "the inner membrane" may include the inner envelope of plastids.

Reviewer #3

(Remarks to the Author)
While the authors have addressed most of my queries about the manuscript I still have major issues regarding the mechanistic interpretation that they present towards the end of the paper and particularly in Fig. 5. In the absence of any additional computational and experimental data they do not have sufficient evidence in my opinion to propose the full mechanism presented in Figure 5. The clear novelty of this work is the unusual position of the propionate group. I agree that the authors should state that this indicates that the propionate is likely to play a major role in the reaction mechanism which

has certainly never been shown before. However, they have no evidence for the order of electron and proton transfers based on the EM structures presented in this work. I also do not know what they mean by an 'exotic -COOH- intermediate'. A PchlideH- species has already been established in previous work (ref 51 for example) rather than a radical intermediate. Have they not considered the possibility of concomitant H-transfer to the propionate and on to the C17 double bond. Such concomitant H-transfers were proposed by Taylor et al (doi:10.1111/febs.17025). The possible inclusion of the active site Cys is also discussed by Taylor et al and this is not mentioned in the current manuscript. Also, is the distance from the NADPH to the C17-C18 bond still consistent with an intial electron transfer to this bond? I can understand that the distance is likely to be too large for H-transfer but electron transfer could still be possible? In my mind this would seem much more plausible than their proposed electron transfer to the propionate, which would already be negatively charged at physiological pHs. In the absence of any additional mechanistic data I recommend that the authors dilute the mechanistic interpretation presented in Fig 5. I would suggest that they hypothesise that the 'hydride' is transferred via the propionate to the C17-C18 bond but that the exact order of electron / proton / H atom transfers remains to be fully elucidated. This in itself is novel and noteworthy but still leaves the full mechanistic understanding open to further studies.

Open Access This Peer Review File is licensed under a Creative Commons Attribution 4.0 International License, which permits use, sharing, adaptation, distribution and reproduction in any medium or format, as long as you give appropriate credit to the original author(s) and the source, provide a link to the Creative Commons license, and indicate if changes were made.
In cases where reviewers are anonymous, credit should be given to 'Anonymous Referee' and the source.
The images or other third party material in this Peer Review File are included in the article's Creative Commons license, unless indicated otherwise in a credit line to the material. If material is not included in the article's Creative Commons license and your intended use is not permitted by statutory regulation or exceeds the permitted use, you will need to obtain permission directly from the copyright holder.
To view a copy of this license, visit https://creativecommons.org/licenses/by/4.0/

We would like to sincerely thank all reviewers for their careful reading of our manuscript and for their constructive comments and suggestions. We genuinely appreciate their attention even to minor issues in the text, as well as their insightful questions. Their thoughtful and thorough feedback has significantly improved the clarity, consistency, and overall quality of the manuscript. We believe that the revised version represents a substantial improvement and presents our findings in a clearer and more accessible way. We are grateful for the time and effort the reviewers have invested, which we feel has meaningfully strengthened the manuscript.

Below, we provide a detailed point-by-point response to all comments and suggestions raised by the reviewers.

Reviewer #1 (Remarks to the Author):

The manuscript Structures of LPOR-Chlide complexes reveal the structural basis of membrane remodeling and photocatalysis presents interesting structural results describing several different LPOR filament architectures from plants using single-particle cryo-EM. Unlike previous studies, this work focuses on Chlide-LPOR in complex with NADPH. The authors show that the LPOR dimer serves as the fundamental building block of the filaments and analyze the periodic deformation of lipid bilayers associated with different filament architectures. Furthermore, based on the high-resolution structures, the authors propose a reaction mechanism consistent with their structural observations. The cryo-EM data appear to be of good quality and are potentially important.
However, at present, the structural novelty of this work are not sufficiently clearly defined. In particular, the manuscript does not adequately distinguish the newly identified filament architectures from those previously reported, including earlier work by the same authors. A clearer comparison with prior studies is essential to establish what is genuinely structurally new and what confirms or extends existing models.
In addition, the proposed reaction mechanism, while plausible, is not experimentally validated in this study. Therefore, either additional supporting evidence should be provided, or the mechanistic interpretation should be presented more cautiously as a proposed model rather than a definitive mechanism.
Overall, the work has the potential to make an important contribution, but its novelty and mechanistic conclusions need to be more clearly articulated. With improved clarification the significance of the findings would be easier to assess.

Major points:
The authors must show a 3D model (e.g. schematically) in the introduction (Either main figure or supplement) where the reader can properly see the current model of the PLB/cubic phase 3D structure of the etioplast. It is very hard to imagine this from the text only. It is known from reference 9 and reference 10 how these organize. It should be shown schematically and it should be specially shown how the LPOR is involved in the organization of the 3D model of the cubic phase/prolamellar bodies. It is unclear for the reader how the proteins are involved in this formation especially because the lipids are trapped into the filament. How is this incorporated into a 3D PLB? This model should then later reappear in the last figures and it should be adapted by the structures found in this paper.

We thank the reviewer for this important comment. However, at present, the organization of LPOR within the three-dimensional structure of the prolamellar body (PLB) remains unknown. The references indicated by the reviewer do not resolve this question. One of the references, our previous work, describes in vitro–determined assemblies, whereas the other, the cryo-electron tomography study shows filaments associated with the PLB but does not resolve their arrangement within the cubic phase itself. Notably, the authors of that study also emphasized

that they were unable to determine the organization of LPOR within the 3D cubic phase of the PLB.

Moreover, work by Kowalewska and colleagues indicates that the PLB is not a simple three-dimensional arrangement of filaments with a constant diameter. Instead, the PLB resembles a minimal surface, in which the diameter of tubular connections varies continuously—being thicker near junctions and thinner in between. This suggests that the organization of subunits within the PLB is variable and cannot be described as a regular filamentous assembly.

Given these limitations, we would prefer to avoid including a schematic representation that would necessarily be speculative and not supported by experimental data. Elucidating the organization of LPOR within the PLB, both in vitro and in native systems, is currently the subject of our ongoing work. We have different hypothesis on the architecture of LPOR within the cubic phase, however, we do not yet have sufficient evidence to propose a reliable model. Nevertheless, a simplified representation of the PLB is shown in Fig. 1D.

Which filaments were known what was not known etc. also how do the filaments described in this paper relate to the filament described before in that model? Then it is also better to judge what exactly is the novelty of this study compared to what was published before.

We added a novel Extended Data Figure 3, when we compare novel assemblies with the one described previously.

Fig 1F: There needs to be a control micrograph. Here it only shows after 10 min the filament is stable but where is the reference micrograph where it would show that with low NADPH concentration it should disassemble or partially disassemble.

We did perform a control with 2 uM NADPH illuminated for 10 minutes however, it is hard to show the absence. Below is a representative micrograph for this condition. Small fragments are visible that we interpret as membrane fragments partially covered with protein. It cannot be excluded that larger fragments of a membrane released from the filaments did not attach to the grid therefore are not seen. We preferer not to include in the manuscript the micrograph shown below to avoid over interpretation of the data, for example the false conclusion that the during the disassembly small pieces of the membrane are released form the filaments. We are still investigating the mechanism of the filament formation and disassembly focusing on the membrane geometry during these processes.
We modified the part of the Results section (P6L23-24) adding the information the no filamentous oligomers were observed in the illuminated sample supplemented with 2uM NADPH.

30 uM AtPORB, 5 uM Pchlide, 2 uM NADPH, 60 uM OPT	
dark	Illuminated for 10 minutes

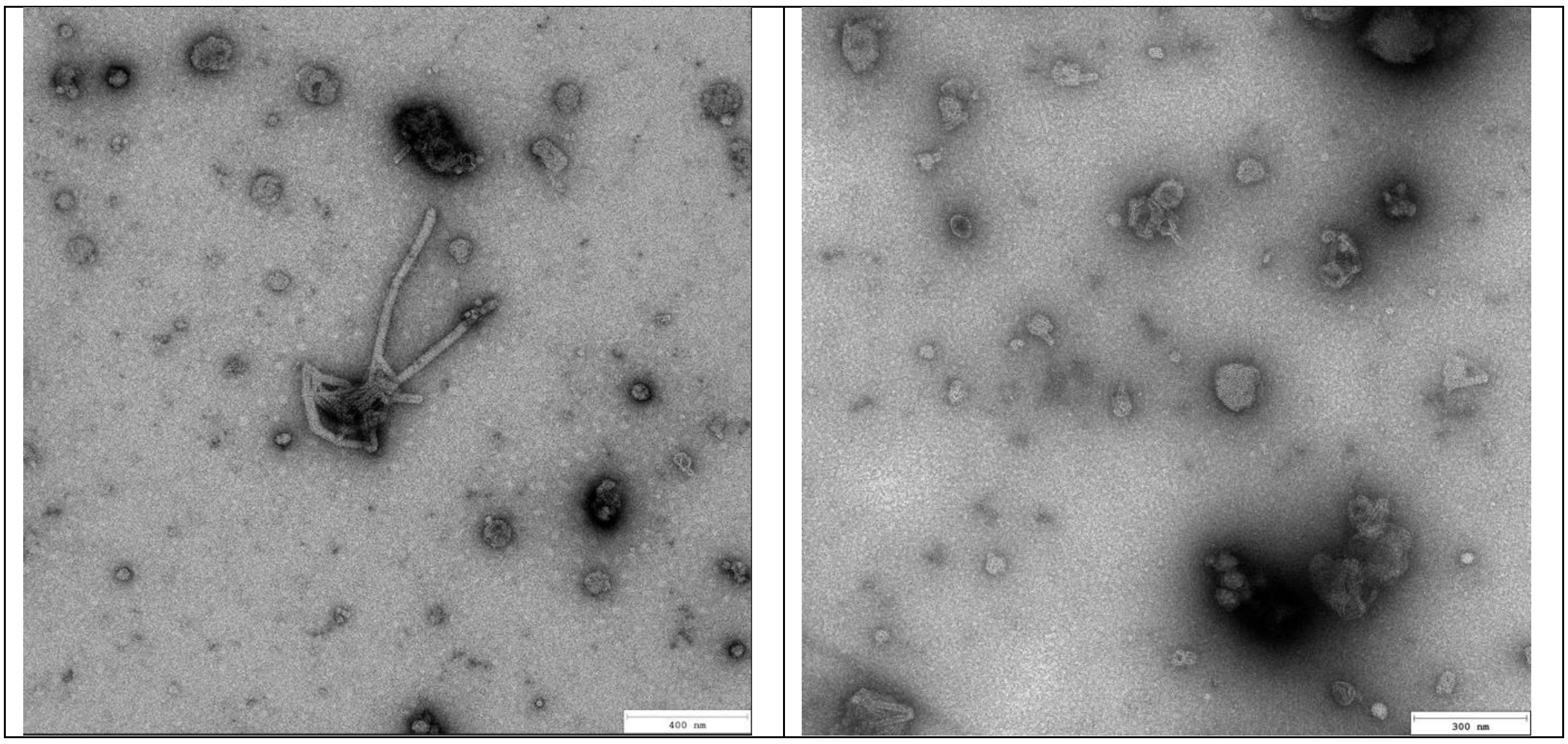

The section and Fig 2D of LPOR assembles into a variety of oligomeric states must be much better described in terms of protein chain composition. How many protein chains form each different filaments what is the packing how does it relate to the filament thickness etc. For example the dimer could be highlighted in the helical filaments then it should show how many dimers in a full rotation or rise twist and pitch directly labelled on each filament. Equally for the ring filaments, as it is already described in the text how many dimers are forming it, it should show it for example by rotating 90 degrees to look down the axis and then to count the number. In Line 211 onwards It says 4 and 3 and 1 strings of dimers. What does it mean? Please highlight those in the figure and label. Also a comparison with previously determined filaments of Pchlide should be shown in supplement.

We modified the part of Results section to better describe the assemblies (P8L1-7). Additionally, we added a novel Extended Data Figure 3, in which we highlighted a representative dimer and a string of dimers in each assembly, according to the reviewer's suggestions. We included the number of subunits per turn, but in the case of these oligomers, calculating the protein chains (subunits) is impossible, since it depends of the length of the filaments. It is better to describe the architecture providing the number of a linear dimer chains (that we call a string of dimers) that form a given assembly (in the case of helical complexes) or the number of dimers in a ring (in the case of stacked-ring complexes). We did not include the top-down views, as they are shown in Fig. 3A but we included the previously determined filament in the Extended Data Figure 3.

Fig 4 ABC: These panels are very hard to understand especially for the definition of alpha and beta angle. These angles need to be better shown in context of model. Where exactly are the alpha and beta in the model. Also the many squares shown with many lines are unclear and confusing.

We added a new Extended Data Fig. 7, in which we show in more detail the angles alpha and beta. We modified the text (P13L15-29) so the origin of this geometric representation is better described. We also modified the Fig. 4D (the one with many squares) and changed is legend. We believe that now this part of the manuscript is easier to understand.

Also it is unclear why only the model with this interaction in panel E F G H are shown only for HF23 and RF23. Any justification for that?

Only the interaction within models HF-23 and RF-23 are shown in detail, because these assemblies exploit all three different oligomerization interfaces (discovered so far), depicted in the Fig.4FGH. The other models utilize the same interactions but in different combinations, as explained in the text and Fig. 4J. We added a novel Extended Data Figure 6 to show that the oligomerization interfaces are nearly identical within the HF-type filaments, and within RF-type filaments. We also modified the respective part of Results section to justify our choice (P14L10-12).

In line 327, 335, and 329 it says mirror-symmetric interaction/arrangements. This is very misleading because there are no mirror symmetries possible in proteins. This whole chapter is very difficult to understand and should be simplified substantially.

The reviewer is correct. We changed "mirror-symmetric" to "symmetric" and re-write the chapter. Now, with additional Extended Data Fig. 6 and 7 we believe that this part of the manuscript is easier to understand.

Extended data Fig 5 should not claim novel reaction mechanism because it is not confirmed it is rather proposed. Also it would be great to see the mechanism that was proposed previously to this study side by side as comparison and why the old mechanism would fail based on the structure. This mechanism should be confirmed by some independent method or very downweighted in claim and said this is a proposed alternative mechanism.

We modified the title of the Figure (now it is Extended Data Fig. 9) to indicate that it is a proposed mechanism, according to the suggestion.
There have been at least three different mechanism proposed within the last 4 years by: Silva and Cheng (10.1021/acscatal.1c05351), Johannissen et al (10.1021/acscatal.2c00866) and Liu et al (10.1016/j.jcat.2025.116158). We recently compared two of these mechanisms in our previous study (Pesara et al 2024, DOI: 10.1039/d4sc00923a). It would be challenging to accurately interpret and reconcile the data presented by other groups into a single detailed mechanistic scheme, particularly given that multiple, distinct contradictory mechanisms have been proposed. None of them included the role of the propionate group in mediating the electrons and proton transfer from NADPH to Pchlide.
We modified the part of the Discussion to underline the novelty (P20L14): the involvement of the propionate group. We are examining this mechanism with QM/MM methods, but this is on-going research and will be a part of different manuscript.

Minor points:
Line 45: It says most models adopt helical or stacked ring forms whereas one forms a string of dimers not seen before. It is unclear to the reader if this "not seen before" is referring only to LPOR or in general. Does this mean the other filaments were seen before in other cases (not LPOR)? This needs to be properly written, what exactly is new and what exactly was not seen before.

We referred to the segmented helical assembly (SF-25), which represents a previously undescribed structural organization among protein oligomers. We modified this part of abstract accordingly.

Line 51: Again confusing with what was written before. It said one string dimer in line 46. Now it says about several different string architectures. Is it now one or many different structures? Again this must be much better phrased and explained so that the readers are not confused.

In the line 46 we referred not to one string, but one of the assemblies determined in this study. In line 51 we claim that the strings of dimers can interact via three distinctive oligomerization

interfaces, so there is no contradiction. We modified the lines 46-47 to avoid confusion (P2L15-16).

Line 66: Should mention that LPOR + Protochlorophyllide + NADPH + light ⇒ Chlorophyllide with references

This information is written in the Introduction, in P3L30-31. We added a suitable reference according to the suggestion.

Line 67: No reference for LPOR involved in cubic phase remodelling

We added a suitable references according to the suggestion.

Line 74: "whose main lipid component is the non-bilayer-forming monogalactosyldiacylglycerol (MGDG)" MGDG are forming bilayers in those paracrystalline structures or not? If they do then it would be better to remove non-bilayer forming because it suggests they are not forming bilayers in the cubic phase but they likely do. This non-bilayer forming refers to isolated MGDG.

We modified this fragment according to the suggestion. The revised version reads*: whose main lipid component is monogalactosyldiacylglycerol (MGDG), which inherently favors non-bilayer phases.*

Line 76: "Thus far, a few crystal structures of cyanobacteria …" Should be changed to Several crystal structures …

Text was modified according to the suggestion.

Line 78: However, the cryo-EM structure of the … : remove However because it was used in the sentence before.

Text was modified according to the suggestion.

Line 79: Should be revealed the binding pocket with its substrate bound

We modified the text according to the suggestion. The revised version reads: *revealed the binding pocket with the substrate bound*

Line 85: redshifts the pigment from ? to 655 nm at 77K …

We modified the text according to the suggestion. The revised version reads: *The lipid binding redshifts the pigment emission from ~647 nm to ~655 nm at 77 K*

Line 90: double bond 17–18 for the D ring of the tetrapyrrole

We modified the text according to the suggestion. The revised version reads: *After exposure to light, LPOR uses NADPH to reduce the C17–C18 double bond in ring D of the tetrapyrrole substrate (…)*

Line 98: Please rephrase to "Immediately after the product (Chlide) formation" for clarity

We modified the text according to the suggestion.

Line 104: rephrase: which is associated with subsequent esterification of Chlide by chlorophyll synthase, that completes Chl synthesis.

We modified the text according to the suggestion.

Line 105: … what ultimately leads to PLB…. Should be … which ultimately leads to PLB

We modified the text according to the suggestion.

Line 122 : Fig1A: I do not see the shaded areas which represent the standard deviation

This is because it is very small.

From Line 120 onwards: Here it describes the monitoring of the fluorescence spectra temperature dependent. In the methods it is however not clear how this is performed. First seedling is grown in dark. Then measurements occur one by one I suppose. First 80K, illumination, recording, then next temperature from the same sample, illumination recording etc. It says namely: For each target temperature after the initial 80 K measurement (without prior illumination), the sample was illuminated for 1 min with saturating white LED light (> 6000 μmol photons m^{-2} s^{-1}) and then dark-incubated for 1 min at the target temperature. Is the phrase without prior illumination now referring to 80K point? It is confusing. Is it first illuminated at 80K then measured and then equilibrated to the next temperature then illuminated again, then 1 min dark, then measured? Or is it illuminated only once? The authors should write that in a more precise way.

We modified this part of Materials and Methods to avoid confusion and misinterpretation, according to the suggestion.

Line128: Chlide or Pchlide?

Chlide – the text is correct. At 160 K temperature, nearly no fluorescence from Chlide nor photoactive Pchlide was detected, due to the formation of non-fluorescence intermediate.

Line 134 use NADPH instead of dinucleotide throughout the manuscript to be consistent

When we refer to 'dinucleotide', we mean either NADPH or $NADP^+$. In this context ('which resembles emissions of Chlide:LPOR complexes lacking the dinucleotide cofactor in vitro'), we refer to the Chlide:LPOR complex after release of $NADP^+$ and prior to binding of NADPH. We modified the text which now reads: *(…) lacking the dinucleotide <u>(either NADPH or NADP+)</u> cofactor in vitro (…)*

Line168 Fig 1E not F

We thank the reviewer for pointing this out. The typo has been corrected in the text.

Line 171 Fig 1F not E

We thank the reviewer for pointing this out. The typo has been corrected in the text.

Line 177 It says to elucidate how LPOR accommodates both Pchlide and Chlide we perform

helical reconstruction of Chlide LPOR NADPH OPT but what about the Pchlide? Rephrase for example remove Pchlide if in this study you did not investigate it.

We investigated Pchlide binding in our previous cryo-EM study. We agree with the reviewer that this sentence may be misleading. We modified the text according to the suggestion (P7L25).

Line 201 18,466 movies not 18466 videos

We modified the text according to the suggestion.

Extended Fig 1C: Please mention if this is in vitro reconstituted sample in the caption. What does it mean complexes of different composition, clarify in detail.

The reviewer is correct: Extended data figure 1C is based on in vitro reconstituted complexes. By “different composition” we mean: either NADP+ or NADPH and Pchlide or Chlide. The revised version reads: *The fluorescence emission spectra of in vitro reconstituted complexes of LPOR (15µM) in complex with NADPH/NADP+ (200 µM) and Pchlide/Chlide (5 µM), in the presence of OPT lipids (400 µM), with emission maxima corresponding to complexes differing in their bound dinucleotide (NADPH or NADP⁺) and pigment (Pchlide/Chlide).*

Figure 1B: It is not clear what those curves mean since there is no explanation in the caption of the color code etc. Also shaded areas I cannot identify. It needs to be explained better what this panel exactly means. Also there is this Shibata shift twice depicted. In vivo and in vivo. Is it meant to be in vivo and in vitro please clarify.

The reviewer is correct: the caption of panel B lacks important information. We extended the caption. The shaded area may be difficult to see since it is small – the replicates are quite similar. Shibata shift is depicted twice: once on the plot showing the emission maxima of the components, the other time on the plot showing the components intensity. Panel B shows only data based on in vivo measurement, that is why it is depicted twice. The revised caption now reads:
Gaussian deconvolution of selected spectra, showing the experimental data with fitted components, as well as the emission maxima and intensities of the fitted components. Experimental spectra are shown as solid lines in a green–yellow color scale reflecting illumination time. Dashed yellow and violet lines indicate the two Gaussian components, and the solid black line represents their sum. Shaded areas represent standard deviation between biological replicates.

Fig 1C: In the text it says the shift is composed of two different components. In the figure 1C please mark the components. Is it meant that the emission spectra shifts from 690 nm to 681 nm as the 2 components. If yes then better not use the word components because it suggests that it is somehow a sum of two components. Please rephrase.

We thank the reviewer for bringing this to our attention. This helped us identify an error in the referencing to the figure in the text, which was unfortunately not detected prior to submission. The figure underwent multiple revisions, during which this labeling inconsistency was introduced. We apologize for this oversight. The part of the text referred to by the reviewer corresponds to Fig. 1B, which shows that the spectra are composed of two Gaussian components. We corrected the panel references to Fig. 1 throughout the text. The description now correctly corresponds to the figure.

Extended Data Fig 1AB: what is the difference between the two? Simply replicates? In any case mention what is what exactly.

These are indeed replicates. We added this information in the figure caption.

Extended data figure 2: Please provide all angular distributions.

We provide a 2D histogram of particle orientations (azimuth vs. elevation), which directly visualizes the angular distribution of the dataset in Extended Data Figure 2L.

Fig 1D. This is unclear diagram: It needs explanation. The NADPH is used in the reaction and NADP+ will be produced. Does NADP+ bind or is it unable to bind please clarify. Then all of a sudden NADPH drops during Shibata shift. No explanation is given why. Is there any process that uses NADPH?

The diagram shown in Fig. 1D is a model of PLB formation and disassembly that is proposed based on the observations from this study and other literature data. The reviewer is correct: NADPH is used in the reaction and NADP+ is be produced after the illumination. NADP+ biding is complicated - we tested 13 LPOR isoforms from 6 species for NADP+ binding in the presence of either Pchlide or Chlide; in a presence of PG, lipid mixture resembling native lipids or with no lipids at all (Gabruk et al 2025, DOI: 10.1016/j.jbc.2025.108261). Most isoforms bind NADP+ and Pchlide, but NADP+ can be easily displaced by NADPH (Ogrodzinska et al, 2024, DOI: 10.1016/j.plaphy.2024.108935). The NADP+ binding in the presence of Chlide seems to be worse than that in the presence of Pchlide; for most of the isoforms in vitro. In vivo however, the high NADPH concentration allows fast displacement of newly formed NADP+ in the binding pocket, as can be seen in Fig. 1A, but we have more evidence for this process now, from multiple species, measured in intact seedlings.
The drop in NADPH concentration 30 minutes after the illumination was shown experimentally by Mapleston et al. so it coincides with Shibata shift. Since it is unknown what triggers the Shibata shift, we speculate it can be triggered by NADPH concentration drop caused by some other process. Our in vitro experiment (Fig. 1E) confirms that LPOR disassembles faster when NADPH concentration is low.
We modified the caption to underline that the model is based on previous studies and that Discussion section provides more details.

Fig. 2 D: It is hard to understand what these + signs mean. Please clarify in detail in the caption what was done. I suppose the RF-21 RF23 and RF25 were averaged. To obtain RDA. If so please give justification to do this in the text and also make clear in the caption that the RF-21 RF23 and RF25 were averaged. Are they really the same to allow averaging? Also add particle number for each subset

The reviewer is correct that RD-A was obtain by averaging RD-21, RD-23, and RD-25, as explained in Materials and Methods. Since all of these maps represented the same stacked-ring architecture, we tried to merge these datasets and the resulting map (RD-A) had improved resolution. The particle number for each dataset is provide in Extended Data Tables1-9 – we induced this information in the revised caption to this figure.

Extended Data Table 3, 5, 6, 7: Model validation is insufficient in terms of Ramachandran outliers and allowed region. The expectation value is 98, 2, 0. For example 93% with 5.75% allowed is energetically unfavorable. Please fix this.

We have improved the models as suggested by the reviewer. This process was particularly time-consuming and consumed most of the time required for the revision, as we aimed to ensure the highest possible accuracy. For RF-23 and RF-25, we were not able to reach 98% favored residues in the Ramachandran plot; the values were 96.01% and 96.97%, respectively, which is a considerable improvement. Given the size and complexity of these maps, which comprise more than 40 subunits, we believe this represents the best achievable refinement under the current conditions.
The rest of the models have at least 97% of favored residues.

We thank the reviewer for highlighting this point. The models have now been improved and we believe they represent the best quality that can be achieved given the size and complexity of the system.

Line 223: Confusion. You show 9 maps 7 filaments and then it says we have 11 maps. Please clarify. Either only mention 9 or say specifically each map and why are the others not shown in Fig 2?

We are sorry for the confusion but both the numbers and the figure 2 are correct.
In Fig. 2 we show 11 maps: HF-23, HF-25, HF-29, RF-21, RF-23, RF-25, SF-25, RD-21, RD-23, RD-25, and RD-A. All of them were submitted to EMDB. Based on these we build 9 models: HF-23, HF-25, RF-21, RF-23, RF-25, RD-21, RD-23, RD-25, and RD-A

Fig 3D please show density as primary proof for lipid and water and pigment

The density of Chlide and MGDG are now shown in Fig. 3. Additional densities are now shown in novel Externed Data Figure 4A.

Fig 3A please label outer and inner leaflet

The figure was modified according to the suggestion.

Fig 3 E and F should be one panel? In the caption the E is missing. In the text as well the panel E is not called. Also the panel is way too small. Maybe move into supplement and make it much larger and label it properly so that the reader can actually see what is shown.

The reviewer is correct, the panel is too small. We created a novel Externed Data Figure 4, in which we now show this panel, as panel B.

Line 311 This subtitle is not very informative. Please change more towards a combination of three different interaction modes between dimers defines the higher oligomeric string formations of filaments

The subtitle was changed to *Filament architecture is defined by at least three distinct interaction modes.*

Line 314: It says: two dimers can interact at least in two distinct ways …. (Fig 4A). In Fig 4A however it does not show two distinct ways. At least it is hard to see from that. Please improve this.

According to the previous suggestions of the reviewer, we re-write this part of the results section.

Line 316: The square with a subunit in each corner. Please clarify where the corner is placed in the text? It seems from the figure it is placed always in different position of the subunit.

Should it not be placed every time in the same exact position? If not define the position of the corner properly and justify the choices.

For this visualization, we always used alpha carbon atom of the residue Q331, which is directly "below" magnesium ion of the pigment. We included this information in the revised text (P13L20-21). However, the resulting geometric representation (i.e. angles) is the same, regardless of the chosen reference point, as long as it is consistent.

Line 315: …the square is nearly planar … It is not clear what this means please rephrase and explain in detail what it means

This part has been rephrased. Additionally, a novel Extended Data Figure 7 now helps to better visualize the geometry behind the architecture of LPOR filaments.

Fig 4 panel B This panel should show the interactions. But it does not show any dashed lines that would point to this. Also it is better to make two panels side by side with RF23 and indicate the differences if any. Please remove hydrogens they make the figure hard to understand

The oligomerization interface, that allow the formation of the string of dimers, shown in panel B, is driven mostly by hydrophobic interactions. No specific interaction can be drawn in Fig. 4B. We added this information in the main text (P13L17-18) and removed hydrogens, according to the suggestion.

Fig 4 panel E G H, again no interactions are shown. Please show the interactions with dashed lines and also remove the hydrogens. What are these transparent models in the background. Remove because they are also very confusing.

We removed hydrogens, added the interactions, and removed the transparent models – according to the suggestion. The transparent models in the background were the respective oligomerization interfaces from either HF- or RF-type assembly, for comparison. Now, the panels are much less confusing. We thank the reviewer for these suggestions.

Fig.5: Again please remove hydrogens they are not very useful. Also it is not consistent because there are no hydrogens shown for water. In addition sometimes they are even in the wrong orientation. For example T230 forms a hydrogen bond with ligand but the hydrogen points opposite direction.

Figure 5 was modified according to the suggestion.

Fig 5 D the C17–C18 bond is not planar this is misleading please show it with planarity

In Figure 5D, we present a schematic representation of the reduction of the C17–C18 bond, illustrating the transition from a planar to a non-planar geometry, indicated by the addition of hydrogen atoms. To avoid any ambiguity, we have clarified in the figure caption that this panel shows a schematic representation.

Line 372 2x).)., remove one

The typo was corrected.

Line 457: FLU should be written the FLU (FLUORESCENT protein)

The text was modified according to the suggestion (P19L18)

Line 472: VIPP1 should be explained what it is in 1–2 sentences.

We added more information on VIPP1 in revised text, according to the suggestion (P19L32-34).

Line 437: In the results part it is said in Line160 that it resembles the Chlide:LPOR spectra without NADPH bound. Here it says Chlide is released. Please clarify what is released in which order. It is confusing. Is not the release of NADPH responsible for the Shibata shift? Please clarify.

The reviewer is correct that this part is confusing and inconsistent. Shibata shift is a term reserved for the shift in fluorescence emission maximum and should not be used in this context. The revised text now reads: *The eventual release of Chlide likely requires…*

Line 481: Please change: A Novel Reaction Mechanism Consistent with Previous Observations to something more like Proposed mechanism for the …
The whole paragraph is written as if the mechanism is kind of figured out, and now only we need to study some more details. However this is not the case, as suggested before either experimental verification and validation need to be provided or the whole paragraph should be written more speculatively and downweighted.

According to the reviewer's suggestion, we modified the title of this part to *A Novel Putative Reaction Mechanism Consistent with Previous Observations*, and included the information that the proposed mechanism requires further experimental validation (P20L11-12).

Line 541 to 543: Although referencing the old studies is good also to spell it out in shorter form what was done here. This is true for all references in the methods. Please briefly describe what was done not just referencing back.

We added a Methods section in Extended Data file in which we briefly describe LPOR production, Pchlide purification and Chlide production.

Line 560: All scripts in this manuscript must be provided in the supplement material

The compressed archive containing the raw data and scripts are now attached as Extended Data File 1.

Line 562: Again please describe what was done not just reference back

We added a Methods section in Extended Data file in which we briefly describe sample preparation and negative staining protocol.

Line 569: Please clarify why such high amount of Arginine is needed. Could it lead to artifacts in the filament formation?

We note that a high arginine concentration is required to prevent filament aggregation. Establishing these conditions was an essential part of protocol development and required extensive optimization. This approach was previously described and used in our earlier study (Nguyen et al, Nature Plants), in which we demonstrated that arginine does not affect the fluorescence emission spectra.

Line 585 18466 should be 18,466

The text was changed according to the suggestion.

Line 591: Mention the word “total dose” which is 40 $e^-/Å^2$

The text was changed according to the suggestion.

Reviewer #2 (Remarks to the Author):

In this mauscript, Gabruk et al. revealed the molecular structures of LPOR-Chlide-NADPH complexes associated with the lipid membrane. Their findings provide novel insights into the reaction mechanism of LPOR, and surprisingly suggest the limited structural changes in LPOR after the photoconversion of Pchlide. These observations lead us to reconsider current models of the LPOR reaction and subsequent remodeling of PLB.
Although this manuscript includes intriguing data and arguments, I am concerned that some of the authors' interpretations may go beyond the data presented, particularly in the following two aspects: (1) The relationship between the LPOR structure and PLB disassembly, and (2) how the structure identified here relates to LPOR function in mature chloroplasts rather than in etioplasts (or etio-chloroplasts). I strongly recommend that the authors reconsider these points to make their manuscript more coherent and convincing.

Major points
1. Lines 119 and 419 and their related sections. I think the authors' data are insufficient to conclude that Chlide:LPOR:NADPH complex formation or NADPH concentration affects the morphological changes of PLBs. I understand that spectral changes in vivo coincide with PLB dissociation and the persistence of LPOR oligomers after light exposure, however, the current data do not indicate in which conditions the PLB structure was disassembled. To clarify this causal relationship, it would be beneficial to perform in vitro experiments using different NADPH concentrations. Moderating the interpretation may be another option. The title and the last sentence of the abstract should also be reconsidered in this regard.

We thank the reviewer for this comment. The experiment suggested by the reviewer is, in fact, already presented in Figure 1E. In this experiment, we analyze the position of the chlorophyllide emission maximum during and after the reaction at different NADPH concentrations in vitro. As expected, at lower NADPH concentrations, the emission maximum shifts more rapidly towards the blue region characteristic of chlorophyllide–LPOR complexes lacking a bound dinucleotide.

In addition, under low NADPH conditions, we were not able to detect any filamentous assemblies by electron microscopy (please, see the response to reviewer 1). This observation further supports our conclusion that NADPH is required to stabilize the oligomeric state of the enzyme. Additionally, Ryberg and Sundqvist (1988, 10.1111/j.1399-3054.1988.tb00589.x) showed that PLB retains its ultrastructure after the illumination when the sample is supplemented with NADPH. We now added this information in Discussion section.

Importantly, we agree with the reviewer that the overall structure of oligomers appear largely unchanged before and after photoconversion. However, we would like to clarify that this apparent structural similarity does not exclude the presence of functionally important conformational dynamics. In particular, following photoconversion, the rapid (within 1 second after the illumination, as seen is Fig. 1AC) release of the dinucleotide bound deeply within the protein must require a transient conformational change to allow its exit (please see the Results section P15L30-34). When NADPH is present at sufficiently high concentrations, it can subsequently rebind, within the first 10 seconds after the illumination (as seen in Fig. 1AC), promoting a return of the enzyme to a closed conformation, which in turn facilitates re-oligomerization.

2. Lines 238, 266-271 and 464-479. The authors claim that LPOR oligomers affect the membrane structure due to their interaction with MGDG. This may be true, however, doesn't the same apply to LPOR oligomers with Pchlide, given that the ternary complex structures are largely unchanged before and after photoconversion of Pchlide? I think that that the alternation of membrane structure cannot be explained solely by the interaction between LPOR and MGDG, but may also involve additional proteins like CURT1 or other lipids like SQDG or PG, which have charged head groups. As the Extended Figure 3 showing the similarity between Pchlide:LPOR;NADPH and Chlide:LPOR:NADPH is very important, I recommend that the authors include it in the main figure.

The reviewer is correct: a similar mechanism of membrane remodeling applies to complexes containing both Pchlide and Chlide. Our in vitro experiments demonstrate that LPOR alone is sufficient to remodel lipid membranes into both filamentous assemblies and cubic phase–like structures resembling the prolamellar body, as previously described (Ogrodzinska et al.). Importantly, the data presented here demonstrate that multiple types of membrane deformations can be induced in MGDG-rich membranes (Fig. 3BC). While the interaction between LPOR and MGDG plays a key role, interactions between LPOR subunits within oligomers also contribute significantly. These protein–protein interactions impose structural constraints that generate directional forces on the membrane, leading to periodic deformations. Since only LPOR was present in our reaction mixtures, these effects can be attributed directly to the intrinsic properties of LPOR and its ability to both interact with lipids and self-organize into higher-order assemblies.

In native systems, interactions with additional proteins are likely to further contribute to the overall membrane architecture, and we fully agree with the reviewer on this point. However, the involvement of additional proteins alone does not explain the mechanism by which membrane reorganization occurs.

Here, we propose a coherent, testable, and predictive molecular mechanism for how membrane remodeling can arise from protein organization. Specifically, our data suggest that membrane deformation is driven by a combination of LPOR–lipid interactions and the intrinsic geometry of LPOR oligomers. In this framework, oligomerization is not merely a consequence of membrane binding, but an active driver of periodic membrane deformation. Within this model, additional proteins may modulate, rather than define, the remodeling process—for example, by altering the spacing between dimer strings or the angles at which these strings propagate, thereby tuning the resulting membrane curvature and architecture.

Consistent with this view, we have clarified in the Results section which now reads:
Such a coordinated deformation of the membrane is possible because <u>within the oligomers</u> each LPOR dimer interacts with two MGDG molecules constraining it at defined angles and positions.

The reviewer is also correct regarding the role of PG and SQDG in LPOR activity. As we demonstrated, these anionic lipids promote NADPH binding, most likely by lowering the energetic barrier between the apo and holo states of the enzyme. In this way, they facilitate the transition toward the NADPH-bound, catalytically competent conformation, as described in: Gabruk et al. 2017 DOI:10.1042/BCJ20170047; Ogrodzinska et al 2024, Fig.3, DOI: 10.1016/j.plaphy.2024.108935).

3. Throughout the manuscript, the discussion related to PLB formation, PLB disassembly, and chlorophyll biosynthesis in mature chloroplasts appears to be mixed. For instance, lines 274-283 discuss the PLB formation based on data from Chlide:LPOR:NADPH complexes. The

effects of photosynthesis discussed in lines 446-455 cannot be directly applied to understand the process in etioplasts or the very beginning phase of etioplast-chloroplast differentiation. Also, LPOR oligomers and PLBs are specific to etioplasts, thus regulatory mechanism of LPOR in mature leaves (lines 462 and 503) may be largely different from those observed in dark conditions. Please reconsider if the current data truly support the proposed mechanism of PLB formation or chlorophyll biosynthesis under continuous light conditions (without PLB).

At this point, we respectfully disagree with the reviewer. In our study, we used the AtPORB isoform, which is expressed both in etiolated seedlings and in mature leaves. The catalytic mechanism of LPOR, and the substrate/product binding constants are intrinsic to the enzyme and are not expected to depend on the tissue context. Moreover, LPOR activity is not restricted to etioplasts but also occurs in mature leaves, where chlorophyll biosynthesis is ongoing. In this environment, NADPH concentrations can vary substantially, providing conditions under which different functional states of the enzyme may be populated. The reviewer is correct that LPOR oligomers with Pchlide are specific to etioplasts, but what about the LPOR oligomers with Chlide? We believe that the data presented in this study suggest that Chlide:LPOR:NADPH complexes can form in mature leaves if NADPH concertation is high enough and the membranes with MGDG are available.

Importantly, we do not claim that large oligomeric assemblies, such as those observed in PLB, are present in mature leaves. Rather, we propose that LPOR oligomerization can occur in mature leaves under conditions of sufficiently high NADPH concentration. This distinction has now been clarified in the revised manuscript, which now reads:

The results presented here show that LPOR:NADPH forms stable complexes with both Pchlide and Chlide, but only the Chlide-bound complexes remain stable upon illumination. <u>This suggests that LPOR oligomers with Chlide may also form in mature leaves during the day, although likely at a smaller scale due to lower enzyme abundance.</u> These complexes can effectively trap the chlorophyll intermediate, thereby linking Chl synthesis to photosynthetic activity: elevated NADPH concentration generated by photosystems can pause the flux at the terminal step.

Minor points

1. Line 67. Please clarify the evidence that the cubic phase is required for chloroplast development.

We thank the reviewer for pointing this out. We agree that the original wording was not optimal and have revised the sentence accordingly. The revised version now states: (…)*into a cubic phase <u>involved in</u> chloroplast development.*

2. Figure 1 B and C. Please explain what the different colors indicate. In Fig. 1C, I failed to understand the meaning of x-axis. Please clarify.

The reviewer is correct that the descriptions of both panels lacked important information. We have therefore extended the figure legend to clarify these points. Panel C integrates information from two complementary types of experiments, combining temperature-resolved measurements with kinetic data. This combined approach allows us to follow the full sequence of events including Chlide:LPOR:NADP$^+$ formation, NADP$^+$ release, and subsequent NADPH binding, up to Shibata shift.
Additionally, we modified Fig. 1C to indicate which data points correspond to temperature-dependent experiments and which to kinetic measurements.

3. Figure 2. Do the authors have any suggestions about which size of LPOR filaments are similar to those observed in etioplasts?

We thank the Reviewer for this question. At present, we do not have a definitive answer. However, previous studies by Kowalewska and co-workers have demonstrated that the PLB is not simply a regular three-dimensional arrangement of tubes with a constant diameter. Instead, based on extensive analysis of TEM micrographs from etiolated seedlings of different plant species, these authors proposed that the diameter of the membrane connections varies continuously between lattice vertices: being thinner in the middle and thicker closer to the vertices. The diameter range in within 20-30 nm range for the tubes in PLB, so comparable with our structures.

This organization resembles a minimal surface-like geometry, where curvature is continuously redistributed across the structure rather than being localized at discrete points. Such an arrangement suggests that the PLB is a highly dynamic and geometrically constrained membrane system. While it remains challenging to rationalize how such a geometry could be maintained solely by the enzyme, one possibility is that periodic membrane deformations induced by protein–lipid interactions contribute to stabilizing this unique architecture.

4. Lines 177-179 and 282-283. Explaining the purpose of next experiment at the end of sections is somewhat unusual. It would be more straightforward to put these sentences at the beginning of the next section.

The text was modified according to the suggestion.

5. Line 513. Triticum aestivum should be written in italics.

The text was modified according to the suggestion.

I hope my comments are helpful for the authors.

We thank the Reviewer for the careful reading of our manuscript and for the insightful, thought-provoking comments and suggestions.

Reviewer #3 (Remarks to the Author):

The article by Gabruk and co-workers, entitled 'Structures of LPOR–Chlide complexes reveal the structural basis of membrane remodeling and photocatalysis' presents new cryo-EM structures of complexes of the light-dependent enzyme LPOR in complex with the product of the photoreaction Chlide. The LPOR enzyme is crucial for both chlorophyll biosynthesis and chloroplast development but crucial structural information on the substrate and product complexes has been lacking. This paper builds on previous cryo-EM work by the same authors where they presented structures of the LPOR-Pchlide (i.e. substrate) complex. Here, the improved resolution of the LPOR-Chlide complexes has provided important information on how these complexes shape the photosynthetic membranes, as well as new insights into the catalytic mechanism of the enzyme. The novelty of the work definitely arises from the higher resolution structural data provided by the elegant cryo-EM data, which appear to be very well carried out. However, some of the interpretations discussed are not completely new and the authors seem to have omitted quite a singificant chunk of older and newer literature. Hence, I have a few concerns that need to be addressed prior to publication:

1. The data and findings presented in figure 1 are not new and the molecular events following photocatalysis have been known for more than 30 years. Some of the older literature can be found in a review by Lebedev and Timko in 1998 (DOI: 10.1023/A:1006082119102) but studies by Boddi and co-workers on PLBs (e.g. Boddi, B., Ryberg, M., and Sundqvist, C.,1993 Analysis of the 77K fluorescence emission and excitation-spectra of isolated etioplast inner membranes,

J. Photochem. Photobiol. BsBiol. 21, 125-133; Boddi, B., and Franck, F., 1997 Room-temperature fluorescence spectra of protochlorophyllide and chlorophyllide forms in etiolated bean leaves, J. Photochem. Photobiol. BsBiol. 41, 73-82) show very similar results to those presented here. In addition, the sequence of product release and co-enzyme rebinding steps have also been shown in vitro, albeit on cyanobacterial enzymes by Heyes and co-workers (DOI: 10.1021/bi049576h; https://doi-org.manchester.idm.oclc.org/10.1074/jbc.M706098200). In the latter, they even present rate constants for each of these steps. The authors need to state what additional data they have found in their work and how it is significantly different to this previous work. They also need to reference all of this previous work and place their work in the context of these previous findings. In my opinion the novelty of these initial results presented in Fig 1 is the fact that Chlide-LPOR-NADPH still forms large oligomeric complexes (Fig 1E). Do they have similar TEM data in the presence of excess NADP+ for the Chlide-LPOR-NADP+ complexes or in the absence of coenzyme (Chlide-LPOR)?

We thank the Reviewer for this insightful comment and for pointing us to earlier work. We fully agree that our study builds upon decades of research describing the photochemistry of Pchlide reduction and subsequent molecular events. We would like to clarify the specific advances provided by our work.

First, in contrast to the majority of previous studies, which were performed on isolated membranes, homogenates, or in vitro reconstituted systems (including the cyanobacterial systems studied by Heyes D. and co-workers), our measurements were performed on nearly intact, etiolated seedlings containing native prolamellar bodies (PLBs). This allows us to follow the process in a physiologically relevant context, where the local environment, organization, and cofactor availability are preserved. Importantly, cyanobacterial LPOR does not form the characteristic oligomeric assemblies associated with the ~655 nm emission observed in plant systems (as described in Ogrodzinska et al. 2024, DOI: 10.1016/j.plaphy.2024.108935), highlighting a fundamental difference between these systems.
While several previous studies (including those cited by the Reviewer) have indeed investigated intact etiolated seedlings, these experiments typically focused on selected stages of the process or specific spectral features.

In contrast to previous studies, we focused on fluorescence emission of Chlide from intact etiolated seedlings, following the process continuously from the earliest thermally driven steps immediately after photoreduction, extending all the way to the Shibata shift, all measured on the same biological material.

This continuity was essential for a reliable interpretation of the spectral changes. By acquiring a self-consistent dataset, we were able to accurately determine the positions of emission maxima and perform their deconvolution, ensuring that the assignment of individual spectral components is based on internally consistent measurements rather than comparisons across separate experiments, or conditions, or protein variants.

We agree with the Reviewer that several previous studies report observations consistent with our findings. However, the mechanistic interpretation of the Shibata shift and the state of chlorophyllide following photoreduction is not fully unified in the literature. We would also like to note that, to the best of our knowledge, a representation such as that shown in Fig. 1C - capturing the sequential spectral progression comprising an initial blue shift, followed by a red shift, and subsequently the blue shift (the Shibata shift) - has not been explicitly presented in this integrated form in the previous literature. While individual components of this progression have been described, they have typically been reported separately rather than as a continuous, unified spectral trajectory.

In response to the Reviewer's question, we do not observe oligomeric assemblies with NADP+ and Chlide of LPOR in the presence of OPT lipids nor with just LPOR, Chlide and OPT lipids. Instead, we occasionally detect irregular aggregates, whose structural nature remains unclear.

We have now expanded Discussion to more comprehensively reference and contextualize the studies mentioned by the reviewer (P18L3-10).

2. For the cryo-EM work the authors add 40uM Chlide to 28uM enzyme (i.e. close to 1:1). It would seem to me that this would result in a significant proportion of unbound enzyme or just with NADPH bound to the enzyme. Do the authors see any such complexes and how can they be sure that the enzyme is fully bound? Could they also comment on the order in which they mix the components together and does this make a difference? For example what happens if you add the NADPH or the lipids last?

In our spectroscopic experiments, we typically use 15 μM enzyme and 5 μM pigment (Chlide or Pchlide), a ratio that, based on our experimental experience, results in nearly complete binding of the pigment to the enzyme – if the concentration of NADP(H) is sufficient. In the present study, however, our aim was to maximize the formation of higher-order complexes, and we found empirically that increasing the pigment concentration increased their abundance.

We note that under these conditions a fraction of the enzyme is likely to remain unbound. However, based on our understanding of the oligomerization mechanism—where pigment–lipid interactions (e.g., with MGDG) play a key role in triggering assembly—such unbound species (monomers or perhaps dimers) are not incorporated into filamentous structures and are not detected in our cryo-EM analysis, which focuses exclusively on filamentous assemblies. We are therefore confident that the subunits within the observed filaments are pigment-bound.

Finally, the order of component addition was established empirically during extensive optimization of the sample preparation protocol. In our hands, sequential addition of nucleotide, lipids, and finally pigment yielded the most reproducible and well-ordered samples. Although this aspect was not systematically investigated.

3. The conformation of the LPOR-Chlide-NADPH dimers is essentially the same as the previously determined LPOR-Pchlide-NADP+ complexes but can they discuss how these compare to those described for the cyanbacterial system also determined by cryo-EM (https://doi-org.manchester.idm.oclc.org/10.1111/febs.15542). There is no mention of any comparison to this other previous work.

We thank the Reviewer for pointing out the cryo-EM study of cyanobacterial LPOR. We are aware of these results and have also investigated cyanobacterial LPOR in detail (Ogrodzińska et al. 2024 DOI: 10.1016/j.plaphy.2024.108935). In our hands, cyanobacterial LPOR is capable of oligomerization – especially in the presence of either NADPH:Chlide or NADP+:Pchlide ; however, this process requires the presence of PG-containing membranes. Notably, MGDG promotes product release from the complex, in contrast to plant LPOR, where it supports oligomerization with the substrate.

Using negative-stain TEM, we found that cyanobacterial LPOR forms irregular assemblies on PG-membranes that lack a well-defined architecture and are not suitable for high-resolution structural analysis. Consistent with this, the study cited by the Reviewer presents low-resolution maps without accompanying 2D class averages, which limits structural interpretation. The authors also suggest that hydrophobic interactions may drive the formation of elongated assemblies, raising the possibility that these structures reflect non-specific aggregation, due to the lack of membranes present in the reaction mixture.

In addition, our phylogenetic analysis indicates that the oligomerization interfaces required for the formation of ordered string-of-dimer assemblies emerged later in evolution, likely in algae (Gabruk and Mysliwa-Kurdziel 2020, DOI: 10.1042/BCJ20200323). Cyanobacterial variants appear to lack appropriately tuned interfaces for forming plant-like oligomeric architectures.

Taken together, while we cannot exclude that certain cyanobacterial or algal LPOR variants may form more regular assemblies (potentially in a membrane context) we consider that the currently available data do not support a direct structural comparison with the assemblies described here. For this reason, we have chosen not to directly compare our complexes with the volume reported by Zhang et al. (2020).

4. In extended data figure 3C the authors show a comparison with their previous structures and highlight 3 Phe residues which appear to be very close together. Do these interact with the pigment in any way? Can the author comment on the importance of these residues as it has been shown that some of these Phe residues are important for catalysis (https://doi-org.manchester.idm.oclc.org/10.1111/febs.15542).

We agree with the Reviewer that the three phenylalanine residues are located in close proximity to the D-ring of protochlorophyllide and are well positioned to interact with the pigment via hydrophobic interactions. In our computational analysis of the Pchlide binding (Pesara et al., 2024 DOI: 10.1039/d4sc00923a), we indeed found that all three residues contribute to the overall pigment binding energy. Consistently, the study cited by the Reviewer shows that mutations at these positions affect the binding constant (Kd), supporting their role in pigment binding. Similar conclusions were drawn in our work with the use of a double Phe mutant (Pesara et al., 2024 DOI: 10.1039/d4sc00923a), where we showed that these residues contribute to stabilizing pigment binding, and are important for the binding also in the non-photoactive conformation of the apo state.

At the same time, the direct involvement of these residues in the catalytic reaction mechanism remains less clear. While the cited study reports changes in proton and hydride transfer rates upon mutation, a specific mechanistic role for these residues has not been proposed. We consider it likely that the observed kinetic effects arise from altered positioning or orientation of the pigment within the binding pocket rather than from a direct catalytic function of these residues.

In line with this, our proposed reaction mechanism does not assign a direct catalytic role to these Phe residues. Moreover, as shown in our previous work (Pesara et al., 2024), the redistribution of electron density within the pigment upon photoexcitation appears to be largely independent of its precise orientation in the binding pocket and is even preserved in the absence of the protein environment. This suggests that the reaction mechanism primarily relies on intrinsic properties of the pigment, while the protein matrix— including these phenylalanine residues—plays a key role in positioning of the propionate group and stabilizing the substrate rather than directly participating in catalysis.

5. One of the key novelties of the paper that results from the higher resolution of the EM data is the protruding propionate group at the C17 position of the pigment molecule and the implications of this for the catalytic mechanism. In the mechanism shown in Fig 5D and Extended data figure 5 they show that after an initial electron transfer from NADPH there is then a proton transfer followed by another electron transfer (both from NADPH). Previous computational studies from Johannissen et al (ref 13) show that this is actually a H-atom transfer rather than 2 separate steps so the authors should change this accordingly.

The computational work of Johannissen et al (10.1021/acscatal.2c00866) rely on the arrangement of the pigment in relation to NADPH and the “Tyrosine193” (corresponding to

Y276 in AtPORB) that is different than the one we uncovered in our maps. Namely, the pigment orientation is flipped (like a pancake) 180 degrees comparing to the pigment orientation that we proposed here and in our previous cryoEM study (Nguyen et al 2021) which is a major difference. Moreover, Johannissen et at did not place the crucial propionate group of the pigment between the C17=C18 bond and NADPH as it can be seen in our maps.
If it is the hydrogen atom that is being transferred via propionate group,as Jogannissen et al suggest, it would result in exotic $-COOH^{-}$ intermediate. This scenario was not considered in the work of Johannissen et al.
However, the work of the same group (Archipowa et al. 2018) showed experimentally a step-wise mechanism similar to the one we proposed. Namely, after the initial electron transfer, they proposed a proton coupled electron transfer, rather than a hydrogen atom transfer. We modified this part of the discussion and added reference to the work of Archipowa et al.

6. Following on from the above point then I don't think it is correct for the authors to state that this reaction mechanism has not been examined directly before. They now have structural validation of the mechanism presented by Johannissen et al. This work also backs up the work shown by Taylor et al in 2023 (oi:10.1111/febs.17025) where a similar mechanism is proposed. In that paper they also point towards the importance of the Cys residue and the fact that the Tyr is key for pigment binding. The current work should be presented in terms of these latest, more recent findings rather than some of the older mechanistic literature which has since been revised.

We respectfully disagree with the reviewer on this point. Johannissen et al. examined different orientation of Pchlide within the binding pocket. The study by Taylor et al. employed the correct orientation of the pigment, however, neither study addressed the involvement of the propionate group as a mediator of electron and proton transfer. While previous work has suggested potential proton donor, this differs from proposing a detailed, step-by-step reaction mechanism as presented here.
When we state that “this reaction mechanism has not been examined before,” we specifically refer to the proposed role of the propionate group as a mediator of electron and proton transfer from NADPH. To the best of our knowledge, this mechanism has not been previously suggested. We have revised the corresponding section of the Discussion to clarify this point (P20L13-14).

Reviewer #1 (Remarks to the Author):

The manuscript has been improved, and the comments have been adequately addressed. Extended Data Figure 3 is a very nice addition and significantly enhances the clarity of the presentation. I have no further comments.

At this point, we would once again like to sincerely thank Reviewer 1 for their insightful comments and valuable suggestions, which have significantly improved the quality and clarity of our manuscript.

Reviewer #2 (Remarks to the Author):

The authors intensively revised the manuscript and substantially improved it.

We thank the reviewer for kind words.

However, two major concerns remain to be addressed.
1. Relationship between Chlide:LPOR:NADPH complexes and PLB disassembly. I understand that the concentration of NADPH changes the behavior of LPOR complexes after illumination. Nevertheless, this does not directly support the authors' idea that formation of Chlide:LPOR:NADPH complexes causes slow PLB disassembly. I agree with the description on page 18, lines 23-25: "Then NADPH rebinds in the LPOR binding pocket, stabilizing an LPOR oligomers with Chlide and transiently delaying PLB disassembly."

We thank the reviewer for this comment. We are continuing to investigate the PLB disassembly, gathering more and more evidence to support our hypothesis on oligomeric Chlide:LPOR:NADPH complexes delaying PLB disassembly. We are finalizing experiments and preparing another manuscript on the topic.

However, I disagree with the phrases on page 2, lines 29-30, and page 5, line 2, because how the complex affects PLB disassembly is not clarified in the manuscript. Thus, I propose to modify the phrase in the abstract to "our data highlight the close relationship between Chlide:LPOR:NADPH complexes and structural changes of prolamellar bodies." The results shown in the manuscript indicates the importance of NADPH concentration in Chlide:LPOR:NAPDH oligomerization, but its consequences are not yet clarified. A previous study also demonstrated the role of NADPH in delaying PLB disassembly and spectral shift of Chlide, but neither of them proved a causal relationship between Chlide:LPOR:NAPDH complex formation and slow PLB disassembly. I do not object to suggesting such a relationship, but the current wording in the manuscript still sounds misleading for me.

We agree with the reviewer that our data support the formation of the oligomeric Chlide:LPOR:NADPH complexes, but we lack the data uncovering what triggers the disassembly. According to the suggestion we decided to modified the two section of the manuscript, which now reads:

Together, our data highlight the close relationship of Chlide:LPOR:NADPH complexes and the delay of prolamellar body disassembly (…)

Chlide:LPOR:NADPH complex formation may delay PLB disassembly

2. Behavior of LPOR in developed chloroplasts. The authors' idea about LPOR oligomerization in chloroplasts lacks sufficient evidence. It is of course possible, and I think dimerization is very likely in chloroplasts due to its structural features, but there are no data demonstrating oligomer formation under prolonged light conditions.

The reviewer is correct. Our suggestion on the role of LPOR in mature leaves in not a conclusion but a hypothesis.

The Chlide:LPOR:NADPH complexes CAN be formed in chloroplasts, but this remains hypothetical. For such an argument, oligomerization of LPOR in mature leaves should be examined experimentally. In this regard, I strongly recommend that the authors modify (or remove) the following parts: page 2, lines 30-31; page 4, line 22; and page19, lines 2-22.

We decided to modify the fragments of the manuscript to better reflect the speculative nature of this interpretation, according to the suggestion. The modified parts now read:

(…) and open the possibility that these assemblies may have a regulatory function in mature leaves.

(…) and open the possibility that Chlide:LPOR complexes may have a regulatory function in mature leaves

This opens the possibility that LPOR oligomers with Chlide may also form in mature leaves during the day, although likely at a smaller scale due to lower enzyme abundance. These complexes could potentially trap the chlorophyll intermediate, thereby linking Chl synthesis to photosynthetic activity: elevated NADPH concentration generated by photosystems may transiently pause the flux at the terminal step. Recent work further shows that plant LPOR isoforms differ in their affinities for NADPH and Chlide36, implying that plants may be able to tune the rate of Chl synthesis by modulating the expression of LPOR isoforms with distinct biochemical properties. If such a regulatory role exists, it may integrate (…)

Minor point
Page 3, line 11. "The inner membrane" is expected to be rephrased as "the internal membrane", because "the inner membrane" may include the inner envelope of plastids.

We thank the reviewer for this suggestion. We modified the text accordingly.

Reviewer #3 (Remarks to the Author):

While the authors have addressed most of my queries about the manuscript I still have major issues regarding the mechanistic interpretation that they present towards the end of the paper and particularly in Fig. 5. In the absence of any additional computational and experimental data they do not have sufficient evidence in my opinion to propose the full mechanism presented in Figure 5.

We have initiated a comprehensive computational investigation of the proposed reaction mechanism; however, this work is still ongoing and will be presented in a separate manuscript. We agree that we have not generated any additional experimental data to directly support the mechanism. Nevertheless, in our opinion, the experimental findings reported by other groups and cited throughout the manuscript are consistent with our interpretation and provide support for the proposed model.

The clear novelty of this work is the unusual position of the propionate group. I agree that the authors should state that this indicates that the propionate is likely to play a major role in the reaction mechanism which has certainly never been shown before. However, they have no evidence for the order of electron and proton transfers based on the EM structures presented in this work.

We agree with the reviewer that, at present, we do not provide direct experimental evidence for the proposed reaction mechanism. Beside the novel structural information presented in this work, we rely on the previously published biochemical and spectroscopic data. Therefore, we acknowledge that our mechanistic interpretation remains a hypothesis.

We believe that the formulation of mechanistic hypotheses based on experimental observations is a fundamental aspect of scientific research. The role of structural studies extends beyond the description of molecular architecture and includes generating testable models that can explain existing observations and stimulate further investigation. Based on the novel structural information presented here, together with previously published biochemical and spectroscopic data, we propose a mechanistic interpretation that is consistent with the currently available evidence.

To this end, we would like to point out that earlier mechanistic proposals put forward by other groups were also largely based on indirect evidence and were formulated without access to crucial structural information that is now available. In particular, previous studies lacked detailed knowledge of the orientation of the pigment within the active site and the position of the propionate group. Now, based on the structural analysis we identify this group as a potentially major element of the reaction pathway.

I also do not know what they mean by an 'exotic -COOH- intermediate'. A PchlideH- species has already been established in previous work (ref 51 for example) rather than a radical intermediate.

We apologize for using this jargon term. By 'exotic -COOH- intermediate' we mean a somewhat unusual state of protonated carboxyl group with additional negative charge.
PchlideH- is indeed one of the intermediates in our reaction mechanism presented in Ex.data Fig.9 on the penultimate step.

Have they not considered the possibility of concomitant H-transfer to the propionate and on to the C17 double bond. Such concomitant H-transfers were proposed by Taylor et al (doi:10.1111/febs.17025).

We have considered this scenario and we believe that such a mechanism is unlikely for at least two reasons. First, under physiological conditions the propionate group of Pchlide is expected to be predominantly deprotonated and therefore present as a carboxylate. Consequently, it is difficult to envisage how possibly it could serve as a hydrogen donor to the C17=C18 double bond without prior protonation.

Moreover, we were unable to identify such a mechanism in the work of Taylor et al. (FEBS Journal, 2024). The concomitant hydrogen atom transfers proposed in their study involve NADPH, Cys226, and the C18 position of Pchlide, rather than the propionate side chain. To the best of our understanding, Taylor et al. did not propose a pathway involving hydrogen transfer from NADPH to the propionate group followed by transfer from the propionate to the C17=C18 double bond.

Tyr193, Thr145 and Lys193 [[15, 16]], were in close proximity to the propionic acid group at the C17 position of Pchlide and remained at a plausible distance (< 4 Å) for H-bonding interactions over the course of the simulation (Fig. 2A,B). These interactions are proposed to be important to position the Pchlide substrate in the correct orientation for the reduction reaction to occur. (…)

In the discussion section they did not mention the involvement of the propionic group in the reaction mechanism, suggesting that Pchlide orientation may not be a catalytic conformation:

MD simulations (…) showed that the putative catalytic residues, Tyr276 and Ser228 were in close proximity to the propionic acid group at the C17 position of Pchlide and remained at an optimal distance (< 4 Å) for H-bonding interactions over the course of the simulation, whereas Lys279 was in closer proximity to the NADPH coenzyme than Pchlide (Fig. 3A,B). The hydride transfer distance from the pro-S hydrogen at the C4 position of NADPH to both C18 and C17 carbons of the Pchlide substrate was too far for efficient transfer to occur (> 6 Å; Fig. 3C), whilst the hydride transfer angle was also sub-optimal (< 120o; Fig. 3D). Although this implies that the model is not fully optimised or is in a nonreactive conformation, these distances/angles may change slightly upon photoexcitation of the Pchlide molecule.

They indeed considered a simultaneous hydrogen atom transfer, but the one involving cysteine residue (C309 in AtPORB):

In this case, the proposed hydrogen atom transfer, which follows an initially excited state electron transfer from NADPH to the C17-C18 double bond of Pchlide [[10]], could possibly proceed from Cys226 to the C18 position of Pchlide, as the distance and angle of transfer is close to optimal. This would be accompanied by a simultaneous hydrogen atom transfer from the NADPH to Cys226, although it should be noted that there is currently no experimental evidence to support or refute such a mechanism.

The possible inclusion of the active site Cys is also discussed by Taylor et al and this is not mentioned in the current manuscript.

The first report showing experimental evidence for the involvement of Cys309 (numeration for AtPORB) in the reaction mechanism comes from Buhr et al. 2008. We now cite this paper in the Discussion section of the updated version of the manuscript.

Also, is the distance from the NADPH to the C17-C18 bond still consistent with an intial electron transfer to this bond? I can understand that the distance is likely to be too large for H-transfer but electron transfer could still be possible? In my mind this would seem much more plausible than their proposed electron transfer to the propionate, which would already be negatively charged at physiological pHs.

We thank the reviewer for sharing their perspective and for suggesting alternative reaction mechanisms. At present, the available experimental evidence does not allow an unambiguous distinction between all possible mechanistic scenarios. We have therefore presented what we consider to be the most plausible interpretation based on the currently available structural and biochemical data. While alternative mechanisms cannot presently be excluded, the mechanism proposed here is chemically valid. We anticipate that our ongoing computational studies, combined with the novel structural insights presented here, will help to further clarify the reaction mechanism and distinguish between the alternative models proposed to date.

In the absence of any additional mechanistic data I recommend that the authors dilute the mechanistic interpretation presented in Fig 5. I would suggest that they hypothesise that the 'hydride' is transferred via the propionate to the C17-C18 bond but that the exact order of electron / proton / H atom transfers remains to be fully elucidated. This in itself is novel and noteworthy but still leaves the full mechanistic understanding open to further studies.

We thank the reviewer for this suggestion. While we acknowledge that the proposed mechanism remains a hypothesis requiring further computational and experimental validation, we believe that proposing and discussing scientifically valid hypotheses is an important part of interpreting novel structural observations. To address the reviewer's concern, we have revised the Results section (page 15) and introduced more cautious wording (e.g., "may" and "could") to better emphasize the hypothetical nature of the proposed mechanism.
